# Microbiota-derived short-chain fatty acids promote Th1 cell IL-10 production to maintain intestinal homeostasis

**DOI:** 10.1038/s41467-018-05901-2

**Published:** 2018-09-03

**Authors:** Mingming Sun, Wei Wu, Liang Chen, Wenjing Yang, Xiangsheng Huang, Caiyun Ma, Feidi Chen, Yi Xiao, Ye Zhao, Chunyan Ma, Suxia Yao, Victor H. Carpio, Sara M. Dann, Qihong Zhao, Zhanju Liu, Yingzi Cong

**Affiliations:** 1 Department of Gastroenterology, The Shanghai Tenth Peoples Hospital, Tongji University, 200072 Shanghai, China; 2 Department of Microbiology and Immunology, The University of Texas Medical Branch, Galveston, TX 77555 USA; 3 Department of Pathology, University of Texas Medical Branch, Galveston, TX 77555 USA; 4 Department of Internal Medicine, The University of Texas Medical Branch, Galveston, TX 77555 USA; 5 Bristol-Myers Squibb, Princeton, NJ 08540 USA

## Abstract

T-cells are crucial in maintanence of intestinal homeostasis, however, it is still unclear how microbiota metabolites regulate T-effector cells. Here we show gut microbiota-derived short-chain fatty acids (SCFAs) promote microbiota antigen-specific Th1 cell IL-10 production, mediated by G-protein coupled receptors 43 (GPR43). Microbiota antigen-specific Gpr43^−/−^ CBir1 transgenic (Tg) Th1 cells, specific for microbiota antigen CBir1 flagellin, induce more severe colitis compared with wide type (WT) CBir1 Tg Th1 cells in Rag^−/−^ recipient mice. Treatment with SCFAs limits colitis induction by promoting IL-10 production, and administration of anti-IL-10R antibody promotes colitis development. Mechanistically, SCFAs activate Th1 cell STAT3 and mTOR, and consequently upregulate transcription factor B lymphocyte-induced maturation protein 1 (Blimp-1), which mediates SCFA-induction of IL-10. SCFA-treated Blimp1^−/−^ Th1 cells produce less IL-10 and induce more severe colitis compared to SCFA-treated WT Th1 cells. Our studies, thus, provide insight into how microbiota metabolites regulate Th1 cell functions to maintain intestinal homeostasis.

## Abstract (web summary)

T cells play a critical role in intestinal homeostasis, with increasing evidence suggesting a role for the microbiome metabolome in modulating this response. Here the authors show short-chain fatty acids promote IL-10 production in Th1 cells.

## Introduction

Gut microbiota and host immune system maintain a love–hate relationship, undergoing the continuous evolution for co-adaptation. The host immune system coordinates the balance of effector and regulatory immune cells, as well as anti- and pro-inflammatory cytokines in the physical condition through interaction with microbiota. Acumulating evidence suggests that host immune system senses the gut bacteria not only through recognition of the pathogen-associated molecular patterns (PAMP)^1^, but in addition by sensing microbial metabolites, which influence the host immune response in the gut and beyond^2,3^. Bacterial fermentation products, particularly short-chain fatty acids (SCFAs) including acetate (C2), propionate (C3), and butyrate (C4), mediate the effects on host physiology and immunity, regulating the function and differentiation of virtually all immune cell repertoire of gut^4,5^. SCFAs can regulate cell functions either by histone deacetylase (HDAC) inhibition^6–8^, or through the activation of metabolite-sensing G-protein coupled receptors (GPR41, GPR43, and GPR109A)^9–11^. SCFAs have been shown to maintain intestinal homeostasis through protecting epithelial barrier integrity^10,12^, promoting B-cell IgA production^13^, and regulating T-cell differentiation^8,14^.

Although great insights have been obtained into the mechanisms that regulate T-cell differentiation into different effector T-cells, it is still not completely clear how T-effector cells are regulated, which is crucial in controlling intestinal inflammation. Among CD4^+^ T-cells, T-helper (Th)1 and Th17 cells reactive to gut microbiota are central to intestinal homeostasis, although the mechanisms involved are still not completely understood^15–17^. Intestinal inflammation can be inhibited by multiple mechanisms, including T-cell production of IL-10, a key immunosuppressive cytokine which can be produced by T-regulatory (Treg) cells and T-effector cells, which has been proven to play a central role in regulation of intestinal homeostasis and prevention of IBD^18,19^.

T-effector cell production of IL-10 has been considered as a self-limiting mechanism to prevent an exaggerated T-cell response in the intestines as well as in other autoimmune diseases, which otherwise would be detrimental^20^. Polymorphisms in the *IL10* locus confer a risk for IBD, including both ulcerative colitis (UC) and Crohn’s disease (CD)^21–23^, and both mice and humans deficient in either IL-10 or IL-10 receptor (IL-10R) exhibit severe intestinal inflammation^19,22,23^. Interestingly, despite intact IL-10 genes in other cell types, CD4^+^ T-cell specific IL-10 conditional knockout mice develop spontaneous colitis that closely resembles the phenotype in complete IL-10 deficient mice^24^, indicating a crucial role of T-cell-derived IL-10 in inhibiting colitis development. Although great efforts and progresses have been made in understanding IL-10 production during T-cell differentiation, the mechanisms that regulate IL-10 production by differentiated T-effector cells are still unclear. This could be vital for inhibiting colitogenetic T-effector cells and suppressing disease progression, eventually treating the disease.

In this report, we demonstrated that SCFAs promoted IL-10 production of microbiota antigen-specific Th1 cells, which was mediated by GPR43. SCFAs impaired the pathogenic potential of gut microbiota antigen-specific Th1 cells in the induction of intestinal inflammation through promoting IL-10 production by Th1 cells. Mechanistically, SCFAs promoted Th1 cell expression of transcription factor Blimp-1, which is dependent on activation of STAT3 and mTOR. Importantly, SCFAs also promoted IL-10 production by T-cells from humans, including IBD patients, which provides a novel therapeutic potential of SCFAs in the treatment of IBD.

## Results

### Gpr43^−/−^ CBir1 Tg Th1 cells induce severe colitis

**Fig. 1:**
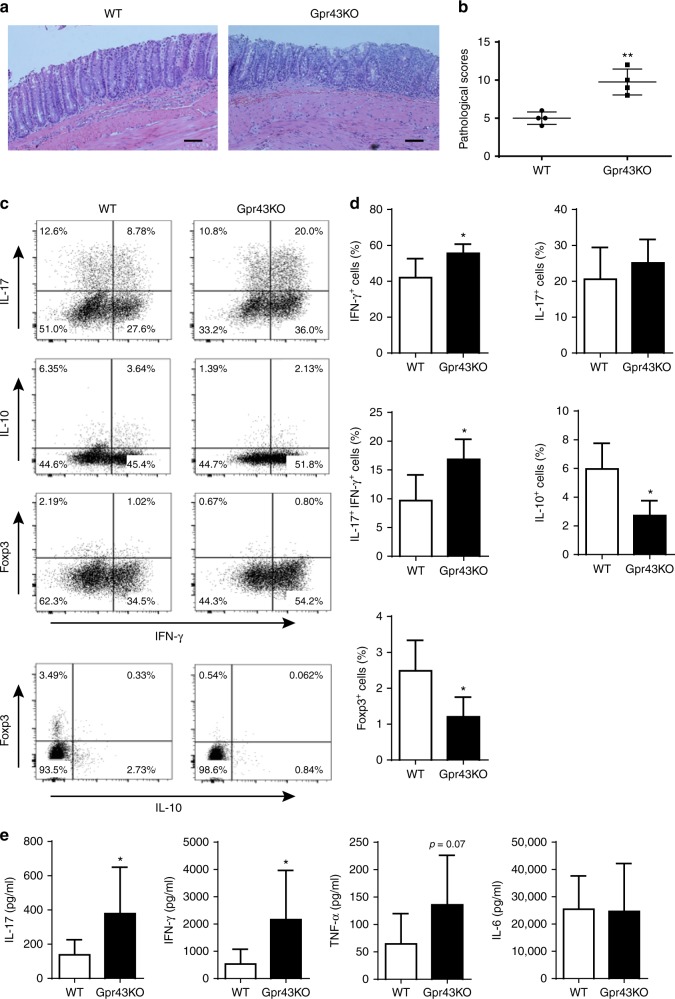
Gpr43^−/−^ microbiota antigen-specific Th1 cells increase pathological potency in induction of colitis. 1 × 10^6^ WT CBir1 (WT) and Gpr43^−/–^ CBir1 (Gpr43KO) Tg Th1 cells were injected i.v. into groups of Rag^−/–^ mice (*n* = 4/group). Six weeks post-T-cell transfer, the severity of intestinal inflammation was assessed. **a**, **b** Colonic histopathology (**a**) and histological scores (**b**) are shown. Scale bar, 100 μm. **p < 0.01 Mann–Whitney *U*-test. **c** LP CD4^+^ T-cell cytokine production and Foxp3 expression were determined by flow cytometry. Plot numbers represent the percentage of CD4^+^ T-cells in the respective quadrants. **d** Bar charts of the percentage of cytokine-expressing CD4^+^ T-cells. **e** Colonic tissues were cultured in the medium for 24 h, and the supernatants were collected for ELISA assay of IL-17, IFN-γ, TNFα, and IL-6. Data are shown as mean ± SD of one representative of three experiments. *p < 0.05 Student’s *t-*test

GPR43 is one of the predominant receptors of SCFAs, and the GPR43-SCFA interaction has been implicated in the maintenance of intestinal homeostasis, in that Gpr43^−/−^ mice develop exacerbated or unresolving intestinal inflammation compared to wide-type (WT) mice in DSS-induced colitis^25^. Although the expression of GPR43 in naïve T-cells is low and SCFAs regulate T-cell differentiation from naïve T-cells mainly through HDAC inhibition, effector T-cells express high levels of GPR43^14,26^. To investigate whether the SCFA-GPR43 regulation of intestinal homeostasis is mediated through T-effector cells, we crossed Gpr43^−/−^ mice with CBir1 TCR transgenic (Tg) mice, which are specific for an immunodominant microbiota antigen CBir1 flagellin^27^. We then generated Th1 cells from Gpr43^−/−^ CBir1 Tg mice and WT CBir1 Tg mice by culture of CD4^+^ T-cells under Th1 polarization conditions with IL-12 for 5-days for two cycles, and then transferred these Th1 cells into Rag^−/−^ mice subsequently. The clinical signs of colitis, including stool consistency and rectal bleeding, were monitored. The mice were sacrificed 6 weeks post cell transfer. The recipients of Gpr43^−/−^ CBir1 Th1 cells developed more severe colitis than the mice receiving WT CBir1 Th1 cells, as evidenced by higher pathological scores (Fig. 1a, b). In addition, we observed that the percentages of IFN-γ^+^ CD4^+^ T-cells, as well as IL-17^+^ IFN-γ^+^ CD4^+^ T-cells, were higher, but levels of IL-10^+^ CD4^+^ T-cells and Foxp3^+^ CD4^+^ T-cells were lower in colonic lamina propria (LP) from Rag^−/−^ recipients of Gpr43^−/−^ CBir1 Th1 cells compared with those from the Rag^−/−^ recipients of WT CBir1 Th1 cells (Fig. 1c, d). There was no significant difference of IL-17 between two groups (Fig. 1c, d). Furthermore, ex vivo culture of colonic tissues showed higher levels of pro-inflammatory cytokines, including IL-17, IFN-γ, and TNFα, in the Rag^−/−^ recipients reconstituted with Gpr43^−/−^ CBir1 Th1 cells compared with the Rag^−/−^ recipients of WT CBir1 Th1 cells (Fig. 1e). There was no difference in IL-6 production between two groups (Fig. 1e).

Collectively, these data demonstrated that Gpr43^−/−^ CBir1 Th1 cells induce more severe colitis, indicating that the SCFA-GPR43 interaction in effector T-cells protects the intestines from inflammation and contributes to the maintenance of intestinal homeostasis.

### SCFAs induce Th1 cell IL-10 production through GPR43

**Fig. 2:**
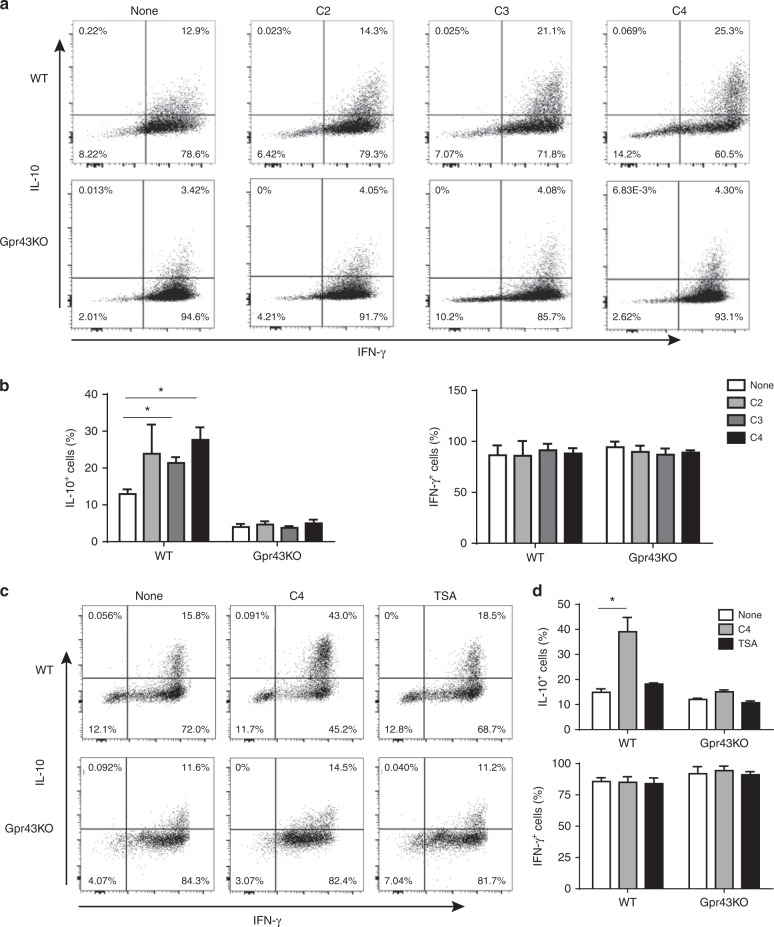
SCFAs induce IL-10 production of Th1 cells through interaction with GPR43. 1 × 10^6^ WT CBir1 (WT) and Gpr43^−/–^ CBir1 Tg (Gpr43 KO) Th1 cells (*n* = 3/group) were cultured with irradiated APC and CBir1 peptide in the presence or absence of acetate (C2, 10 mM), propionate (C3, 0.5 mM), and butyrate (C4, 0.5 mM) for 5 days. **a**, **b** The expression of IL-10 and IFN-γ was examined by flow cytometry analysis. **c**, **d** 1 × 10^6^ CBir1 Th1 cells were stimulated with C4 and HDAC inhibitor TSA (10 nM), respectively, for 5 days. The expression of IL-10 and IFN-γ was examined by flow cytometry analysis. Results are shown as mean ± SD of triplicates from one representative of three experiments performed. *p < 0.05 one-way ANOVA test

To determine how SCFA-GPR43 interaction regulates the function of Th1 cells and inhibits Th1 cell induction of colitis, we generated Th1 cells by culture of CD4^+^ T-cells from WT CBir1 and Gpr43^−/−^ CBir1 Tg mice under Th1-polarizing conditions with IL-12 for 5-days for two cycles. Th1 cells were then treated with acetate (10 mM), propionate (0.5 mM), or butyrate (0.5 mM), for 5 days. Treatment with SCFAs increased the frequency of IL-10^+^ cells in WT Th1 cells (Fig. 2a, b). Interestingly, the IL-10^+^ T-cells were also IFN-γ^+^. In contract, SCFAs did not promote IL-10 production by Gpr43^−/−^ Th1 cells (Fig. 2a, b), indicating that SCFAs promote Th1 cell IL-10 production through GPR43. However, SCFA treatment did not affect expression of IFN-γ, Foxp3, and IL-17 either in WT or Gpr43^−/−^ Th1 cells (Fig. 2a, b, Supplementary Fig. 1a).

Since SCFAs have been shown to promote naïve T-cell IL-10 production during differentiation and Treg cell development, which is mediated by HDAC inhibition^14,31,32^, we then determined whether HDAC inhibitory activity of SCFAs also contributes to increased IL-10 production in differentiated Th1 cells. We treated CBir1 Th1 cells with butyrate or a HDAC inhibitor, TSA (10 nM)^6^, for 5 days. The results of flow cytometry and ELISA demonstrated that while butyrate induced high levels of IL-10 in WT Th1 cells, TSA did not affect IL-10 production in both WT and Gpr43^−/−^ Th1 cells (Fig. 2c, d, and Supplementary Fig. 2), indicating that SCFA induction of IL-10 production in differentiated Th1 cells is mediated by GPR43 but not through HDAC inhibition. To carefully investigate HDAC inhibitory activity in regulating IL-10 production by naïve T-cells and differentiated Th1 cells, we also treated naïve CBir1 T-cells under Th1 differentiation conditions with butyrate or TSA side by side. TSA induced IL-10 production by naïve T-cells under Th1 differentiation conditions (Supplementary Fig. 3), which is consistent with the previous report^14^. Although it has been shown that SCFAs promoted naïve T-cell differentiation into Th1 cells and increased IFN-γ production^14^, we found that SCFA treatment did not affect IFN-γ production by differentiated Th1 cells (Fig. 2a, b). In addition, SCFAs treatment did not affect Th1 cell expression of T-bet, the master transcription factor of Th1 cells (Supplementary Fig. 1b).

**Fig. 3:**
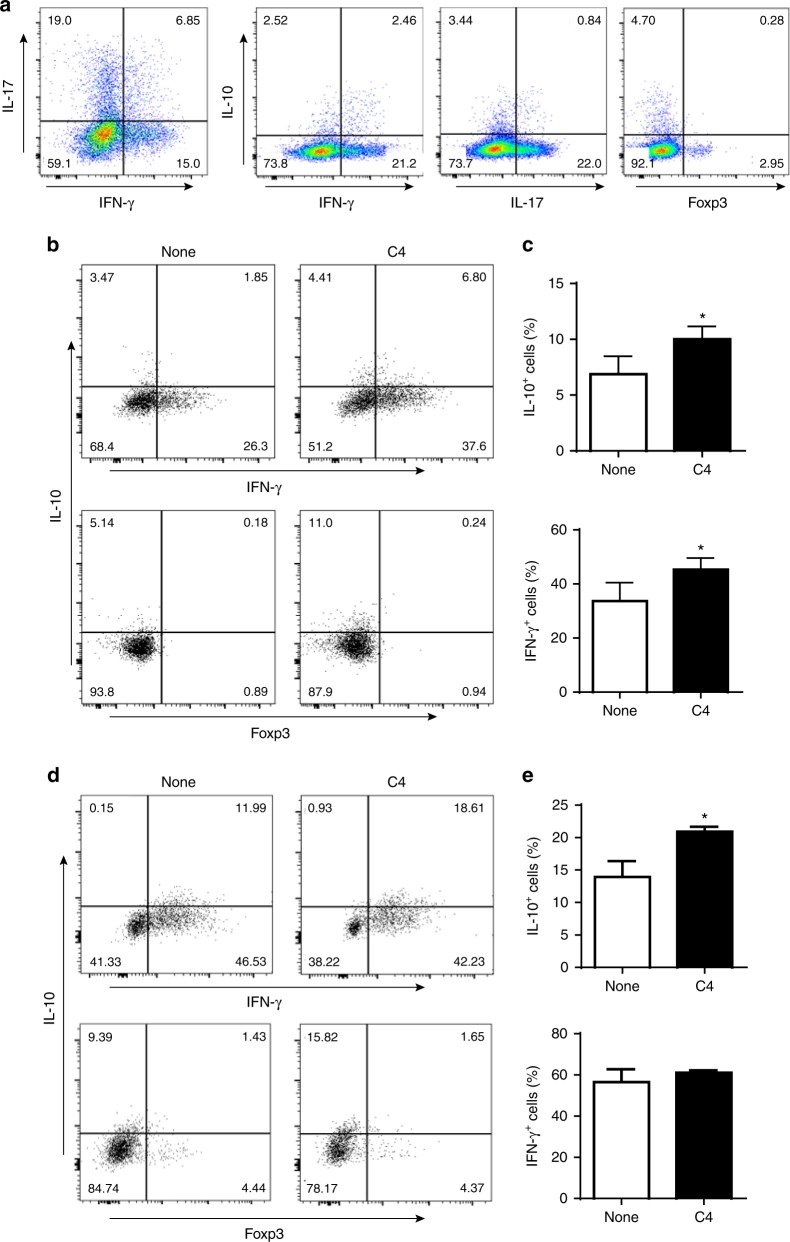
Th1 cells express IL-10 in inflamed mucosal lesions and SCFAs induce T-cells IL-10 production in vivo. **a** 1 × 10^6^ naïve CBir1 Tg T-cells were injected i.v. into Rag^−/–^ mice (*n* = 5). T-cell expression of IL-10, IFN-γ, IL-17, and Foxp3 in lamina propria was examined six weeks after cell transfer when mice developed colitis by flow cytometry. One representative of four experiments performed. **b**–**e** 1 × 10^6^ naïve CBir1 Tg T-cells (**b**, **c**) or differentiated CBir1 Tg Th1 cells (**d**, **e**) were injected i.v. into Rag^−/–^ mice. Groups of 4–5 mice were fed with or without butyrate (C4, 200 mM) in drinking water from the day of cell transfer for 10 days. T-cell expression of IL-10 and IFN-γ in lamina propria was examined by flow cytometry analysis. Both FACS profile (**b**, **d**) and bar charts (**c**, **e**) are shown. Results are shown as mean ± SD of 4–5 mice from one representative of two experiments performed. *p < 0.05 Student’s *t*-test

To explore the significance of IL-10 producing Th1 cells in regulation of colitis in vivo, we transferred naïve CBir1 Tg T-cells into Rag^−/−^ mice. Six weeks later after the recipient mice developed colitis, we assessed IL-10 production by Th1, Th17, and Treg cells in LP of the inflamed intestines. Although all T-cell subsets produced IL-10, around 50% of IL-10^+^ cells were IFN-γ^+^ Th1 cells (Fig. 3a), indicating that Th1 cells are dominant producers of IL-10 among all T-cell subsets following naïve CBir1 Tg T-cell transfer into Rag^−/−^ mice. Thus, Th1 cells may not only serve as an inducer of colitis but also serve as a brake for colitis progression once IL-10 is produced.

To determine if SCFAs are able to promote IL-10 production by Th1 cells and by naïve T-cells in vivo, we transferred differentiated CBir1 Th1 cells or naïve CBir1 T-cells into Rag^−/−^ mice. The recipient mice were fed with or without butyrate in drinking water for 10 days. Butyrate promoted IL-10 production by both naïve T-cells (Fig. 3b, c) and differentiated Th1 cells (Fig. 3d, e), while promoted IFN-γ production in naïve T-cells, but not in differentiated Th1 cells in vivo (Fig. 3b–e). Taken together, these data indicates that SCFAs promote IL-10 production by differentiated Th1 cells through GPR43, but not inhibition of HDAC.

### Low Gpr43^−/−^ Th1 cell IL-10 contributes to excessive colitis

**Fig. 4:**
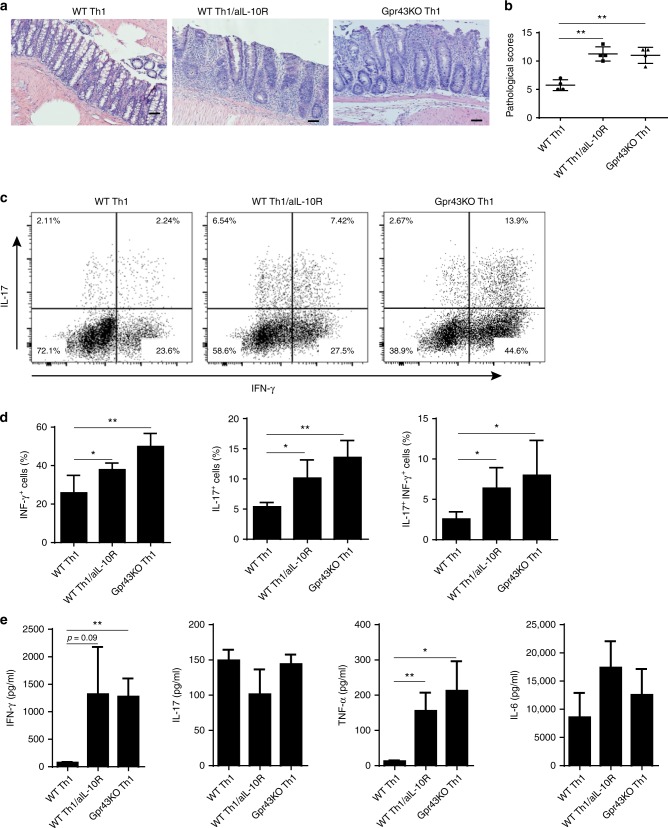
Low IL-10 production in Gpr43^−/−^ Th1 cells contributes to their high pathogenicity in induction of colitis. WT CBir1 (WT) and Gpr43^−/–^ CBir1 Tg (Gpr43KO) Th1 cells (1 × 10^6^/mouse) were injected i.v. into groups of RAG^−/–^ mice (*n* = 4–5/group). One group of mice transferred with CBir1 Th1 cells were administrated with anti-IL10R antibody (clone 1B1.3 A, 25 mg/kg) i.p. weekly. The other two groups of mice were given control IgG. Six weeks post-T-cell transfer, the severity of intestinal inflammation was assessed. **a**, **b** Colonic histopathology (**a**) and histological scores (**b**) are shown. Scale bar, 100 μm. **p < 0.01 Mann–Whitney *U*-test. **c** LP CD4^+^ T-cell cytokine production was determined by flow cytometry. **d** Bar charts of the percentage of cytokine-expressing CD4^+^ T-cells. **e** Colonic tissue cytokine production after 24 h of culture was measured by ELISA. Data are shown as mean ± SD of twelve mice samples pooled from three experiments. *p < 0.05, **p < 0.01 one-way ANOVA test

Since Gpr43^−/−^ Th1 cell induction of severe colitis was accompanied by a lower level of IL-10^+^ T-cells, and SCFAs drastically increased IL-10 production by Th1 cells through GPR43, we next investigated whether the low level of IL-10 production by Gpr43^−/−^ Th1 cells plays a role in exacerbating colitis development. We transferred WT CBir1 Th1 cells and Gpr43^−/−^ CBir1 Th1 cells into Rag^−/−^ mice. A group of Rag^−/−^ mice which received CBir1 Th1 cells were administered with anti-IL-10R antibody (Clone 1B1.3 A, 25 mg/kg) intraperitoneally (i.p.) weekly to block IL-10 signaling, and the other two groups of Rag^−/−^ mice reconstituted with WT CBir1 or Gpr43^−/−^ CBir1 Th1 cells were administered with control IgG. After treatment with anti-IL-10R mAb, the recipient mice that were reconstituted with WT CBir1 Th1 cells developed more severe colitis compared to IgG-treated mice reconstituted with WT CBir1 Th1 cells, at the level similar to that of mice reconstituted with Gpr43^−/−^ CBir1 Th1 cells (Fig. 4a, b). Moreover, IFN-γ^+^, IL-17^+^, and IFN-γ^+^IL-17^+^ T-cells were markedly increased in the LP, and higher levels of IFN-γ and TNF-α were found in the supernatants of colonic organ cultures, from WT CBir1 Th1 cell-reconstituted mice after administration of anti-IL-10R antibody versus control IgG-treated mice reconstituted with WT CBir1 Th1 cells (Fig. 4c–e). Taken together, these data indicate that SCFA-GPR43 interaction in Th1 cells limits excessive inflammation at least partially through induction of T-cell IL-10 production.

### Blimp-1 mediates SCFA induction of Th1 cell IL-10 production

**Fig. 5:**
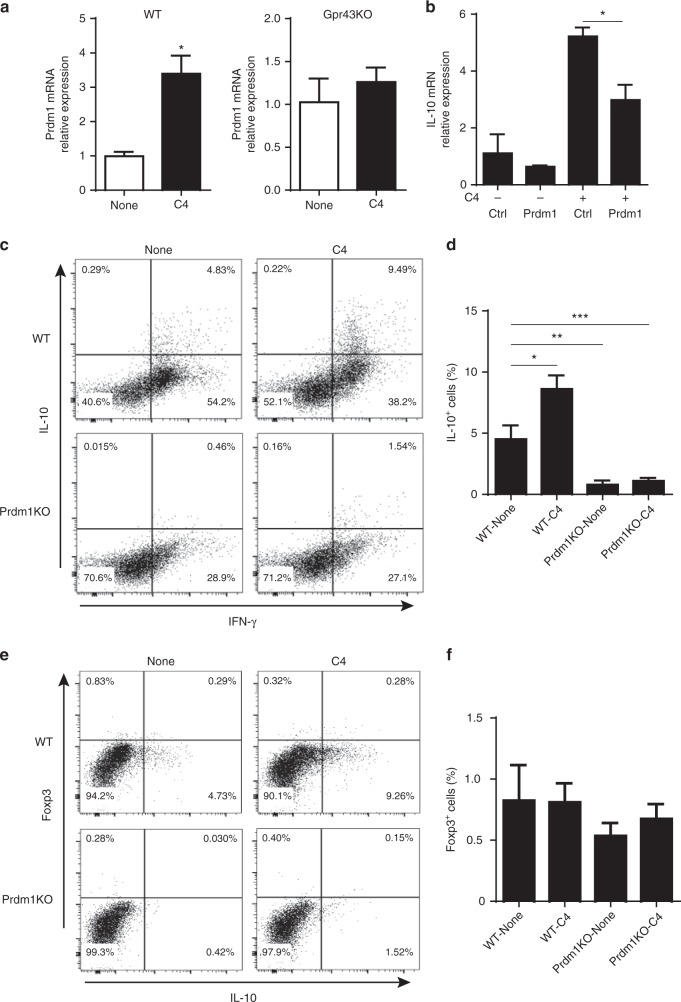
Blimp-1 mediates butyrate induction of IL-10 production in Th1 cells. **a** WT CBir1 (WT) and Gpr43^−/–^ CBir1 Tg (Gpr43KO) Th1 cells (*n* = 3/group) were cultured with APC and CBir1 peptide in the presence or absence of C4 for 5 days. The expression of Blimp-1 was examined by qRT-PCR. **b** WT CBir1 Th1 cells were transfected with Prdm1 siRNA or control siRNA. After 24 h of transfection, the cells were treated with or without C4, and IL-10 expression was determined by qPCR. **c**–**f** WT and Prdm1^−/−^ Th1 cells were cultured with APC and anti-CD3 mAb in the presence or absence of C4. Expression of IL-10, IFN-γ, and Foxp3 was determined by flow cytometry. qRT-PCR results are shown as mean ± SD of triplicates, and FACS profiles are a representative of triplicates from one of three experiments performed. *p < 0.05, **p < 0.01, ***p < 0.001 one-way ANOVA test

Critical roles have been demonstrated for B lymphocyte-induced maturation protein 1 (Blimp-1, encoded by the *Prdm1* gene) in IL-10 production by T-cells, including Th1 and Th17 cells, as well as in T-regulatory cells^28–30^. Furthermore, polymorphisms of Prdm1 have been associated with multiple autoimmune diseases, including systemic lupus erythematosus, rheumatoid arthritis, and IBD^31–34^. We then investigated whether SCFAs promote Th1 cell expression of Blimp-1, which could mediate increased IL-10 production. We were also interested in whether GPR43 mediated SCFA-induction of Blimp-1 in Th1 cells. Treatment with butyrate and acetate increased Prdm1 expression in WT CBir1, but not Gpr43^−/−^ CBir1 Th1 cells (Fig. 5a and Supplementary Fig. 4a). To determine if the HDAC inhibitory activity of SCFAs also plays a role in promoting Th1 cell expression of Prdm1, we treated CBir1 Th1 cells with the TSA (10 nM). Consistently, TSA did not induce expression of Prdm1 in Th1 cells (Supplementary Fig. 4a). All together, these data indicate that SCFAs promote Th1 cell expression of Prdm1 in a GPR43-dependent manner. Since interferon regulatory factor (IRF) 4 has been shown to regulate the expression of Prdm1 and IL-10 in T cells^35^, we then investigated if SCFAs regulated Irf4 expression in Th1 cells. However, butyrate and TSA did not affect the expression of Irf4 in Th1 cells (Supplementary Fig. 4b).

To determine whether increased Blimp-1 mediated SCFA induction of Th1 cell IL-10 production, we transfected Th1 cells with Prdm1 siRNA, which encodes Blimp-1, or control siRNA, then treated these cells with or without butyrate. IL-10 expression was determined by qPCR. As shown in Fig. 5b, control siRNA transfected-Th1 cells produced higher levels of IL-10 upon treatment with butyrate, whereas Prdm1 siRNA greatly compromised butyrate induction of IL-10 expression (Fig. 5b). We also cultured WT and Prdm1^−/−^ Th1 cells from CD4^cre^ Prdm1^fl/fl^ mice in the presence or absence of butyrate. Although treatment with butyrate increased IL-10^+^ cells in WT Th1 cells, the IL-10^+^ cells were greatly reduced in Prdm1^−/−^ Th1 cells (Fig. 5c, d). Butyrate did not induce Foxp3 expression and IL-10 production in Foxp3^+^ Treg cells in both WT and Prdm1^−/−^ Th1 cells (Fig. 5e, f). Put all together, those data indicate that Blimp-1 mediates butyrate induction of Th1 cell IL-10 production.

**Fig. 6:**
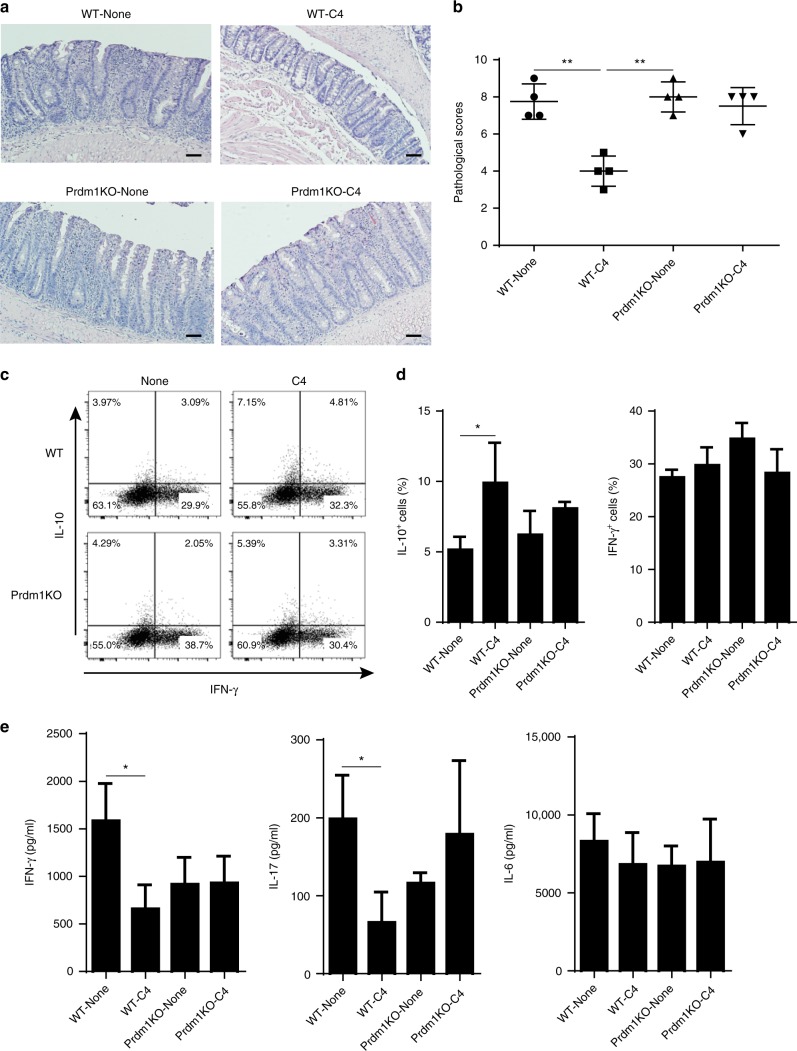
Butyrate inhibits pathogenicity of WT Th1 cells but not Prdm1^−/−^ Th1 cells in induction of colitis. WT and Prdm1^−/−^ Th1 cells were cultured with APC and anti-CD3 mAb in the presence or absence of C4 for 5 days, and then injected i.v. into groups of Rag^−/–^ mice (*n* = 4). Six weeks post-T-cell transfer, the severity of intestinal inflammation was assessed. **a**, **b** Colonic histopathology (**a**) and histological scores (**b**) are shown. Scale bar, 100 μm. **p < 0.01 Mann–Whitney *U*-test. **c** LP CD4^+^ T-cell cytokine production was determined by flow cytometry. **d** Bar charts of the percentage of cytokine-expressing CD4^+^ T-cells. **e** Colonic tissue cytokine production after 24 h of cultures was determined by ELISA. Data are one representative of three independent experiments. *p < 0.05 one-way ANOVA test

To investigate whether Blimp-1 affects SCFA regulation of the pathogenic capacity of Th1 cells in induction of colitis, we generated WT and Prdm1^−/−^ Th1 cells from CD4^cre^ Prdm1^fl/fl^ mice. After treated with or without butyrate for 5 days, we then transferred these cells into Rag^−/−^ mice. The mice were sacrificed 6 weeks post-cell transfer. The Rag^−/−^ recipient mice of butyrate-treated WT Th1 cells developed less severe colitis compared with Rag^−/−^ mice reconstituted with WT Th1 cells, characterized by less epithelial damage, less inflammatory cellular infiltration, and lower pathological scores (Fig. 6a, b). Rag^−/−^ mice which received control Prdm1^−/−^ Th1 cells or butyrate-treated Prdm1^−/−^ Th1 cells developed colitis at similar levels, but more severe than the Rag^−/−^ mice reconstituted with butyrate-treated WT Th1 cells (Fig. 6a, b). In addition, IL-10^+^ T-cells were significantly increased in LP of Rag^−/−^ mice reconstituted with butyrate-treated WT Th1 cells compared with WT Th1 cells-reconstituted recipients (Fig. 6c, d). In contrast, the frequency of of IL-10^+^ T-cells in LP of Rag^−/−^ mice reconstituted with butyrate-treated Prdm1^−/−^ Th1 cells were lower than that in Rag^−/−^ mice reconstituted with butyrate-treated WT Th1 cells, at the levels similar to that in Rag^−/−^ mice with control Prdm1^−/−^ Th1 cells (Fig. 6c, d). Although comparable frequencies of IFN-γ^+^ and IL-17^+^ T-cells were recovered in the LP from all four groups of the recipient mice, the levels of IL-17 and IFN-γ in colonic tissues of the Rag^−/−^ recipient mice of butyrate-treated WT Th1 cells were decreased compared with those from WT Th1 cells-reconstituted controls (Fig. 6e). IL-17 and IFN-γ production in colonic tissues of Rag^−/−^ mice reconstituted with butyrate-treated Prdm1^−/−^ Th1 cells was similar with the mice that received control Prdm1^−/−^ Th1 cells, and colonic IL-6 production was similar in all groups (Fig. 6e). The findings that butyrate-treated Prdm1^−/−^ Th1 cells produced lower levels of IL-10 and were not able to control colitis as effectively as butyrate-treated WT Th1 cells indicate the importance of Blimp-1-mediated SCFA induction of IL-10 production in controlling colitis development.

### STAT3 and mTOR mediate SCFA-induction of Blimp-1 expression

**Fig. 7:**
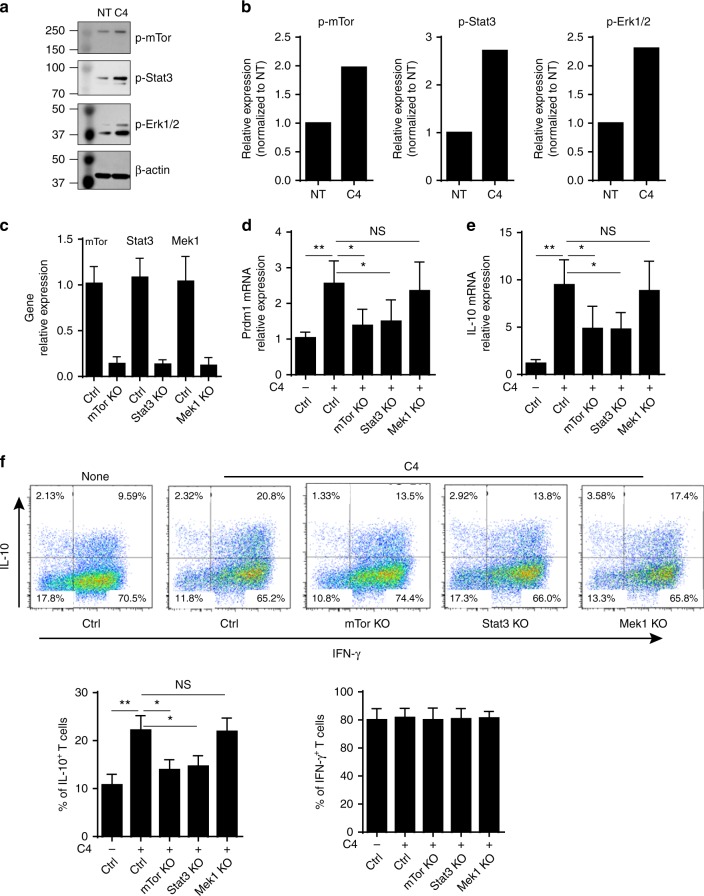
STAT3 and mTOR mediate Butyrate-induction of Th1 cell expression of Blimp-1 and IL-10. **a**, **b** Th1 cells were cultured with anti-CD3 and anti-CD28 mAbs in the presence or absence of C4. The cell lysates were prepared, and Western blot was used to determine p-mTOR (S2448, 15 min), p-SATA3 (Y705, 15 min), and p-ERK1/2 (137F5, 15 min), with β-actin as reference. **c**–**f** Th1 cells were transfected with lentiCRISPR plasmids to knock out Stat3, mTor, and Mek1. After 24 h, the expression of relative genes (**c**) was examined by qRT-PCR. The cells were cultured with anti-CD3 and anti-CD28 mAbs in the presence or absence of C4 (*n* = 3/group). The expression of Prdm1 (**d**) and IL-10 (**e**) was examined at day 2 by qRT-PCR. **f** T-cell cytokine production was determined at day 5 by FACS. Both representative FACS profile and barchart of IL-10^+^ and IFNγ^+^ T-cells were shown. The results are shown as mean ± SD of three samples and are representative from one of three experiments performed. *p < 0.05, **p < 0.01 one-way ANOVA test

We then explored the underlying mechanisms by which SCFAs induce Th1 cell expression of Blimp-1. Activation of STAT3 has been reported to be crucial for the induction of Blimp-1 in B cells and T-cells^35–37^, and the mTOR and MEK-ERK pathways are required for the activation of STAT3^36,38^. Moreover, emerging evidence suggests that SCFAs regulate T-cell differentiation through activating the mTOR pathway^14^. Therefore, we then investigated whether SCFAs upregulate Th1 cell expression of Blimp-1 through potentiating STAT3, mTOR, or MEK-ERK activation. We found that butyrate enhanced phosphorylated-STAT3, mTOR, and ERK1/2 as evidenced by Western blots (Fig. 7a, b). To examine whether the activation of STAT3, mTOR, and MEK-ERK mediates butyrate-induction of Blimp-1 and IL-10 in Th1 cells, we utilized CRISPR technique to knock out Stat3, mTor, and Mek1 in Th1 cells. The efficiency of knockout of these targets was around 80–90% (Fig. 7c). Knockout of Stat3 and mTor significantly inhibited butyrate-induced Prdm1 expression, however, knockout of MEK1 pathway did not affect Prdm1 expression (Fig. 7d). Consistently, knocking out Stat3 and mTor, but not Mek1, decreased IL-10 production in Th1 cells at both mRNA and protein levels (Fig. 7e, f). However, knocking out Stat3 and mTor did not affect Th1 cell IFN-γ production (Fig. 7f). Taken together, these data demonstrated that activation of STAT3 and mTOR pathways are required for butyrate induction of Blimp-1 and, consequently, IL-10 production in Th1 cells.

### SCFAs promote T-cell IL-10 production of IBD patients

**Fig. 8:**
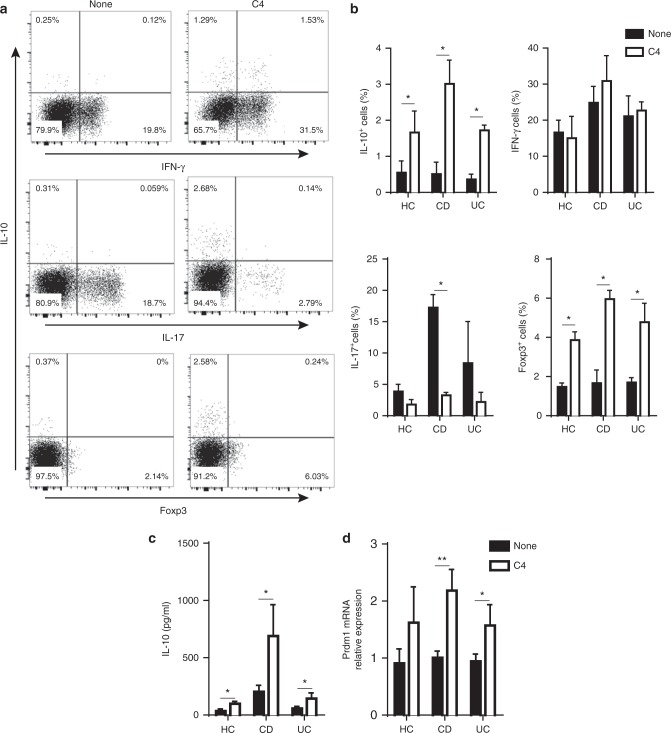
Butyrate promotes IL-10 production by T-cells from patients with IBD. CD4^+^ T-cells from peripheral blood mononuclear cells from IBD patients of CD (*n* = 6), UC (*n* = 6), and healthy individuals (HC, *n* = 6) were cultured with anti-CD3 mAb and anti-CD28 mAb in the presence of C4 for 5 days. **a** CD4^+^ T-cell cytokine production and Foxp3 expression were determined by flow cytometry. **b** Bar charts of the percentage of cytokine-expressing CD4^+^ T-cells. **c** IL-10 in supernatants of cell culture and **d** CD4^+^ T-cell expression of Prdm1 were examined by ELISA and qRT-PCR, respectively. The results are shown as mean ± SD of triplicates and are representative from one of three experiments performed. *p < 0.05, **p < 0.01 Student’s *t*-test

To determine the translational potentials for SCFA induction of T-effector cell IL-10 production, we then investigated if SCFAs regulate IL-10 production by human T-cells, especially from patients with IBD, which will provide potential targets for regulating T-cell functions in IBD patients. We cultured peripheral blood CD4^+^ T-cells, isolated from healthy donors and patients with active CD or UC, with anti-CD3 mAb and anti-CD28 mAb in the presence or absence of butyrate. Consistent with the data from the mouse studies, butyrate promoted IL-10^+^ CD4^+^ T-cells from both healthy donors and IBD patients (Fig. 8a, b, the representative FACs plots data shown were from CD patients). IL-10 production in culture supernatants was also increased by treatment with butyrate (Fig. 8c). Furthermore, butyrate promoted the expression of Prdm1 mRNA in T-cells from IBD patients and healthy controls (Fig. 8d). Interestingly, while butyrate promoted Foxp3^+^ Treg cells, which is consistent with previous reports in mice, it greatly inhibited production of IL-17 in T-cells from both healthy controls and IBD patients. However, butyrate did not affect IFN-γ production (Fig. 8a, b).

### Oral feeding butyrate inhibits colitis induced by DSS

**Fig. 9:**
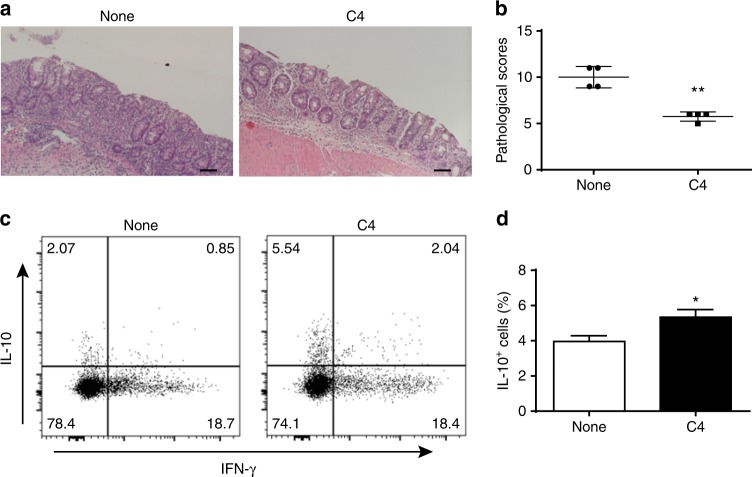
Oral feeding butyrate protects colitis development induced by DSS. B6 mice were administered 1.7% DSS in drinking water for 7 days, followed by 3 days of water. One group (*n* = 4–5) of mice were fed with butyrate (C4, 200 mM) in drinking water from day 0 when DSS was given. **a** Representative images of histopathology in cecum. Scale bar, 100 μm. **b** Histopathological scoring. **p < 0.01 Mann–Whitney *U*-test. **c** LP CD4^+^ T-cell IL-10 and IFNγ production was determined by flow cytometry. **d** Bar charts of the percentage of IL-10-expressing CD4^+^ T-cells. *n* = 4–5 per group per experiment. Data are one representative of 2 independent experiments; *p < 0.05 Student’s *t*-test

To determine whether SCFAs inhibit colitis development, we induced colitis in WT mice through administration of dextran sulfate sodium (DSS). Mice were fed with DSS in drinking water for 7 days and then with drinking water only for additional 3 days. Groups of the mice were fed with or without butyrate at 200 mM in drinking water from day 0. When sacrificed at day 10, we found that the mice fed butyrate demonstrated less severe disease (Fig. 9a, b). IL-10 producing Th1 cells were present in the inflamed colonic tissues of colitic mice, which were further increased by feeding butyrate (Fig. 9c, d).

## Discussion

Accumulating evidence indicates that T-cells reactive to gut microbiota antigens are crucial in mediating the pathogenesis of IBD. Great efforts and progress have been made in understanding the development of those T-cells and mechanisms involved. However, how pathogenic T-effector cells are regulated following differentiation, which represents the T-cells in the inflamed lesions, is relatively unclear. Thus, it becomes important in targeting these pathogenic T-effector cells in treatment of IBD. We demonstrated in this report that SCFAs, produced by gut microbiota as fermentation products of dietary fiber, promoted Th1 cell production of IL-10, thereby inhibiting colitis induced by pathogenic Th1 cells. Different from SCFA regulation of T-cell differentiation, which is mediated by HDAC inhibition^39,14,26^, SCFAs promoted differentiated Th1 cell production of IL-10 through GPR43.

SCFAs regulate the function of immune cells mainly through intracellular targets such as HDAC^6,14,26,39^ and by binding GPCRs such as GPR41, GPR43, and GPR109a^10,13^. SCFAs bind to these GPCRs with varying affinities, and GPR43 recognizes an extensive range of SCFAs, including acetate, propionate, and butyrate^8^. The central role for GPR43 in regulation of intestinal inflammation has been established by the fact that Gpr43^−/−^ mice suffered from more severe DSS-induced colitis^25^. Although naïve T-cells express GPR43 at very low levels^26^, the increased expression of GPR43 in T-effector cells^8,14^ indicated that the SCFA-GPR43 interaction might regulate the functions of T-effector cells. Our current study demonstrated that Gpr43^−/−^ Th1 cells induced more severe colitis compared to WT Th1 cells. The reduced IL-10^+^ T-cells and increased IFN-γ^+^IL-17^+^ T-cells in the LP of mice transferred with Gpr43^−/−^ Th1 cells indicate that the SCFA-GPR43 interaction regulates Th1 cell function, thus providing a potential mechanism whereby Gpr43^−/−^ Th1 cells induce more severe colitis. This argument was further supported by the observation that SCFAs promoted Th1 cell production of IL-10 through interaction with GPR43, rather than HDAC inhibition. An elegant study by Park et al. demonstrated that SCFAs promoted naïve T-cell production of IL-10 during differentiation, which is mediated by HDAC inhibition^14^. Among multiple possibilities behind the different mechanisms by which SCFAs regulate IL-10 production in naïve T-cell undergoing differentiation vs. differentiated Th1 cells, naïve T-cells express GPR43 at a very low level^26^, whereas differentiated Th1 cells express high levels of GPR43^6,14^. We speculate that strong activation of GPR43 by SCFA in differentiated Th1 cells may mask the effect of HDAC inhibitory activity, while HDAC inhibitory activity is dominant in SCFA regulation of naïve T-cells.

Different from previous reports that SCFAs promote naïve T-cell differentiation into IFN-γ-producing Th1 under Th1-differentiaion conditions^14^, SCFAs did not affect IFN-γ production of differentiated Th1 cells. Furthermore, SCFAs did not promote IL-17 and Foxp3 expression by Th1 cells, suggesting that SCFA treatment does not promote Th1 cell conversion into Th17 cells and Treg cells. Among all subsets of CD4^+^ T-cells, Th1 cells have been perceived as the most stable subsets. Although it has been reported that Th1 cells can acquire the phenotype of Th17 cells under inflammatory conditions^40^, unlike Th17 cells, Th1 cells do not convert into Foxp3-expressing Treg cells even under Treg-polarizing conditions or in inflammatory sittings^40,41^. Therefore, secretion of the immunosuppressive cytokine IL-10 by Th1 effector cells is an essential mechanism of self-limitation during inflammation. In fact, Th1 cells are dominant producers of IL-10 among all T-cell subsets in inflamed intestinal tissues of colitic mice (Fig. 3). The central role of increased IL-10 production by SCFA-treated Th1 cells in suppression of Th1-driven intestinal inflammation is further confirmed by the fact that blockade of IL-10 pathway exacerbated colitis induced by butyrate-treated Th1 cells (Fig. 4). More importantly, SCFAs also promoted IL-10 production of human T-cells from healthy individuals as well as from IBD patients, which highlights the translational potential for SCFAs as therapeutic target in treatment of IBD. Indeed, oral feeding butyrate promoted intestinal T-cell IL-10 production and inhibited colitis development upon DSS insults (Fig. 9), further supporting for SCFAs as therapeutic target in treatment of IBD. However, SCFA administration has not been notably successful as a treatment of IBD, although it may have some marginal effects in ulcerative colitis. Among many possible reasons, as short chain fatty acids are readily absorbed by the epithelial cells in the intestinal track, an appropriate dose would be crucial. It has been reported that SCFA mixtures enemas and butyrate enemas had better beneficial effects in patients with UC^42,43^, an appropriate route should also be considered. More importantly, understanding the mechanisms by which SCFAs regulate IBD will provide insights into how to use SCFAs in treatment of IBD. Thus, more mechanistic studies are needed for exploring SCFAs as therapeutic target in treatment of IBD.

The polymorphisms of Prdm1, which encodes Blimp-1, have been associated with autoimmune diseases and IBD^31–34^. Blimp-1 is required for the differentiation of plasma cells as well as for the maintenance of T-cell function and homeostasis^28,44–48^. It is also important for regulating IL-10 production in T-cells. Although Blimp-1 expression and downstream IL-10 production are elevated in the early stage of Th1 differentiation, Blimp-1 expression is repressed at the effector state^47,49^. This is consistent with the transient nature of IL-10 production in highly polarized Th1 cells, whereby Th1 cells could maintain its respective phenotype and function. Our data showed that SCFAs did not only promote Th1 cell IL-10 production, but also increased Blimp-1 expression significantly. The deficiency of Blimp-1 abrogated SCFA-induced IL-10 production in Th1 cells, suggesting that Blimp-1 mediated SCFAs promotion of IL-10 production.

Previous reports suggested that IFN-γ^+^IL-10^+^ Tr1 cells expressed high levels of Blimp-1^49^ and can be derived directly from Th1 cells due to their similar gene expression. These Tr1 cells have the capacity of inhibiting inflammation by producing IL-10^49^. In our study, less severe colitis induced by SCFA-treated Th1 cells compared to control Th1 cells is in line with the increased IL-10 production by Th1 cells. However, adoptively transferred butyrate-treated Prdm1^−/−^ Th1 cells induced colitis at levels similar to control Prdm1^−/−^ Th1 cells in Rag^−/−^ mice, suggesting that butyrate-treated Th1 cells could be endowed with regulatory properties like Tr1 cells in a Blimp-1-dependent fashion. Emerging evidence suggests that Blimp-1 transcription can be triggered by STAT3, which has been identified as an activator of Blimp-1 through binding the Prdm1 response element^35–37^. In addition, the mTOR and MEK-ERK pathways are required for the activation of STAT3^36,38^. Our findings demonstrated that butyrate activated the STAT3, mTOR, and ERK1/2 pathways, and signaled through the STAT3 and mTOR, but not ERK, pathways in Th1 cells to promote expression of Blimp-1 and IL-10.

In summary, our studies revealed that gut microbiota metabolites SCFAs constrain the pathogenic potential of gut microbiota antigen specific Th1 cells to maintain intestinal homeostasis and suppress colitis progression through production of IL-10. Importantly, SCFAs can also promote IL-10 production of human T-cells not only from normal individuals but also from IBD patients, thus, providing a translational potential for treatment of IBD patients.

## Methods

### Animals and models

C57BL/6 and Rag^−/−^ mice were purchased from the Jackson Laboratory, and bred and maintained in the animal facilities of the University of Texas Medical Branch (UTMB). CBir1 TCR Tg mice were bred in the animal facilities of UTMB. Gpr43^−/−^ (Ffar2^tm1Lex^) mice were obtained from Bristol-Myers Squibb^13^, and crossed to CBir1 TCR Tg mice. B6.129-*Prdm1*^*tm1Clme*^*/J* (Prdm1^fl/fl^) mice were purchased from The Jackson Laboratory (Bar Harbor, ME) and CD4^Cre^ mice were purchased from Taconic (Hudson, NY). CD4^Cre^ Prdm1^fl/fl^ mice were generated by crossing Prdm1^fl/fl^ mice with CD4^Cre^ mice. CD4^Cre^ Prdm1^fl/+^ mice were used as controls. The control and experimental mice and recipient mice were littermates and co-housed. All experiments were complied with all relevant ethical regulations, and reviewed and approved by the Institutional Animal Care and Use Committee of the University of Texas Medical Branch. The animal care was in accordance with institutional guidelines of UTMB.

T-cell adoptive transfer colitis: 1 × 10^6^ Th1 cells or naïve CD4^+^ T-cells were adoptively transferred to Rag^−/–^ mice by intravenous injection in the relevant experiments as described in figure legends. The mice were sacrificed at six weeks after T-cell transfer. Dextran sulfate sodium (DSS) colitis: C57BL/6 mice were administrated with 1.7% DSS (Cat#: DS1004, Gojira FC) w/v in drinking water for 7 days, followed by 3 days of water, and were monitored daily.

### Butyrate treatment

The mice were given butyrate (200 mM) in drinking water during DSS treatment from day 0 to day 10.

### Patients

EDTA anticoagulated blood samples (5 ml) were obtained from the patients with active CD, active UC, and healthy controls. All patients were from Department of Gastroenterology, the Shanghai Tenth People’s Hospital (Shanghai, China). The diagnosis of CD and UC was based on clinical, radiological, endoscopic examination, and histological findings. The disease severity was assessed according to international standard criteria such as Crohn’s Disease Activity Index (CDAI) for the diagnosis of CD patients and Mayo scores for UC patients (Supplementary Table 1). All studies were complied with all relevant ethical regulations, and approved by the Institutional Review Board for Clinical Research of Shanghai Tenth People’s Hospital, Tongji University. Written informed consent was obtained from all subjects involved in this study after the nature and possible consequences of the studies were explained.

### Reagents and antibodies

Anti-mouse CD4, IFN-γ, IL-17, and IL-10 antibodies (Cat#: 100544, 505806, 506922, and 505008) were purchased from BioLegend, and fluorochrome-conjugated anti-mouse Foxp3 antibody (Cat#: 17-5773-82) was purchased from Invitrogen. Fluorochrome-conjugated anti-human CD4, IFN-γ, IL-17, IL-10, and Foxp3 antibodies (Cat#: MHCD0412, MHCIFG01, 45-7179-42, 12-7108-82, and 17-4776-42) were purchased from Invitrogen. Foxp3 staining buffer sets (Cat#: 00-5523-00) and Live/dead cell viability assay kits (Cat#: L10119) were obtained from Invitrogen. Recombinant mouse IL-12 (Cat#: 577002) was from BioLegend. Butyrate (Cat#: 303410), acetate (Cat#: S5636), propionate (Cat#: P1880), TSA (Cat#: t8552) were from Sigma Aldrich. Anti-IL-10 receptor antibody (Cat#: BE0050) was obtained from Bio X Cell. The following ELISA assay kits were purchased from Biolegend: mouse IL-10, IFN-γ, IL-17, TNF, IL-6, and human IL-10 antibodies (Cat#: 431414, 430804, 432504, 430904, 431304, and 430604). Lipofectamine RNAiMAX Transfection Reagent (Cat#: 13778100) was purchased from Thermo Fisher Scientific. SMARTpool siRNAs specific for murine Prdm1 and non-targeting siRNA were purchased from Dharmacon. Western blot antibodies against phosphorylated STAT3 (Y705, Cat#: 9145), phosphorylated mTOR (S2448, Cat#: 2971), phosphorylated ERK1/2 (137F5, Cat#: 4695), β-actin (catalog number: 8457), and anti-rabbit secondary antibody conjugated with HRP (Cat#: 7074) were purchased from Cell Signaling Technology.

### CD4^+^ T-cell isolation and generation of Th1 cells in vitro

CD4^+^ T-cells were isolated from the spleens of mice using anti-mouse CD4-magnetic beads (Cat#: 551539, BD Biosciences). To polarize Th1 cells, CD4^+^ T-cells were cultured with irradiated splenic antigen-presenting cells (APCs) and CBir1 peptide, or with anti-CD3 and anti-CD28 mAbs, respectively, in the presence of IL-12 (10 ng/ml) for 5 days for two cycles.

### Isolation of lamina propria cells

The intestines were physically emptied and sliced into 1 cm pieces. Tissue pieces were incubated in PBS containing 0.5 mM EDTA (Sigma-Aldrich) at 37℃ for 40 min under slow rotation, and washed by PBS to remove epithelial cells and intraepithelial lymphocytes. The rest tissues were then cut into 1–2 mm pieces and digested with 0.5 mg/ml Collagenase IV (Cat#: c5138, Sigma-Aldrich) and 2 µg/ml DNase (Cat#: AMPD1, Sigma-Aldrich) at 37 ℃ for 30 min under slow rotation. The supernatants were filtered by 100 µm mesh. The cells were resuspended in 40% Percoll and carefully overlaid onto 75% Percoll. After centrifugation, the interface containing the lamina propria lymphocytes was collected.

### siRNA transfection

siRNA transfection in Th1 cells was performed by using Lipofectamine RNAiMAX Transfection Reagent according to the manufacturer’s instructions. Briefly, 5 × 10^5^ Th1 cells were incubated with 30 pmol siRNA and 3 µl Lipofectamine RNAiMAX transfection reagent in 500 µl OPTI-MEM medium for 6 h in a 24-well plate, followed by replacing with 1 ml normal medium. Transfection efficiency was determined at 24 h post-transfection. Transfected Th1 cells were then cultured with C4.

### Knockout of STAT3, mTOR, and MEK by using CRISPR

The lentiCRISPR vectors (plasmid#: 52961, Addgene) established by the Zhang lab^50^ were used. The design and cloning of the target gRNA sequences was performed as recommended by the Zhang lab GeCKO website (http://www.genome-engineering.org/gecko/). The suitable target sites for gRNA sequence design against STAT3, mTOR, and MEK were identified using the CRISPR design tool software at http://crispr.mit.edu. Cas9-target sites for the indicated genes were designed in http://crispr.genome-engineering.org. Then, synthetic gRNA (ITN) containing target sites were sub-cloned into the lentiCRISPR vector, and transfected into Th1 cells. After 24 h, Th1 cells were cultured as indicated in the text. Transfection efficiency was determined at 24 h post-transfection. Guide RNA oligo sequences for lentiCRISPR are listed in Supplementary Table 2.

### Flow cytometric analysis

The cells were stimulated with PMA (50 ng/ml) and ionomycin (750 ng/ml) for 5 h, with adding 0.7 μl/ml Golgistop (monensin, BD Biosciences) for the last 3 h of culture. After staining of live/dead dye and surface marker of CD4, the cells were fixed and permeabilized, followed by staining with fluorochrome-conjugated anti-mouse antibodies against IL-10, IL-17, IFN-γ, and Foxp3 (1:100). Cells were finally fixed in 1% buffered paraformaldehyde, and quantitated with an LSRII/Fortessa and FACSDiva software (Becton Dickinson, Mountain View, CA, USA). Data were analyzed using FlowJo software. The Gating strategies are shown in Supplementary Fig. 5.

### ELISAs

ELISAs of cytokines (IL-10, IL-17A, IFN-γ, IL-6, TNF) in cells and organ culture-supernatants were performed according to the manufacturer’s instructions. Capture antibodies (1:200) were coated in the plate overnight in 4 ℃. After blocking, samples were incubated for 2 h at room temperature, followed by incubation with detection antibodies (1:200) for 1 h. Subsequently, streptavidin conjugated with horseradish peroxidase was added, and substrate was added after 30 min. Finally, absorbance was measured in an ELISA reader.

### Quantitative real time PCR (qRT-PCR)

Total RNA was extracted with TRIzol reagent and followed by cDNA synthesis. Quantitative PCR reactions were performed by using TaqMan or SYBR green gene expression assays on a Bio-Rad iCycler (Bio-Rad, Hercules, CA, USA), and all data were normalized to GAPDH mRNA expression. All primers used are listed in Supplementary Table 2.

### Histopathological assessment

At necropsy, the cecum and colon were separated and Swiss rolls were prepared. Tissues were fixed in 10% buffered formalin and paraffin embedded. 5 μm sections were prepared and stained with H&E. The severity of tissue damage was quantified by hyperplasia; goblet cell number; crypt abscesses; ulceration; mucosa and submucosa inflamamtory cell infiltration. A score of 0–3, denoting increasingly severe abnormality, was assigned for each of these parameters, and added together as a total histological score. The slides were read blindly.

### Western blot

The total protein was isolated through radio-immunoprecipitation assay (RIPA) with phenylmethanesulphonyl fluoride (PMSF, 1 mM), protease inhibitor cocktail, and phosphatase inhibitors, and the concentration was determined with a BCA Protein Assay kit. Proteins were separated electrophoretically by NuPAGEBis-Tris mini gels (Life Technologies) and probed with mouse primary antibodies (1:2000), followed by incubation with a HRP-conjugated anti-rabbit secondary antibody (1:2000). The bands were detected by enhanced chemiluminescence. All uncropped scans of western blot are shown in Supplementary Fig. 6.

### Statistical analysis

The samples/animals were randomly allocated to experimental groups. Sample and animal sizes were selected based upon our experience in order to achieve sufficient power to detect biologically relevant differences.

Based on whether the data were normally distributed and the number of tested groups for comparison, the levels of significance were determined by appropriate statistical analysis. The nonparametric Mann–Whitney *U*-test was used for assessing pathology scores. Student’s *t*-test was used to determine levels of significance for comparisons between two groups, and one-way ANOVA test was performed to analyze the difference among groups by using Graphpad Prism 5.0 software. Results are shown as mean ± SD; Statistical significance is indicated as follows: *p < 0.05; **p < 0.01; ***p < 0.001.

## Electronic supplementary material

Supplementary information

Peer Review

## Data Availability

The data that support the findings of this study is available from the corresponding author upon reasonable request. All relevant results of this work are available within the paper and supplementary information files.
