# Supplementary material for: Microbiota-derived short-chain fatty acids promote Th1 cell IL-10 production to maintain intestinal homeostasis
Source: Nat Commun. 2018 Sep 3;9:3555. doi: 10.1038/s41467-018-05901-2 (PMC6120873; doi:10.1038/s41467-018-05901-2)
Supplement: Supplementary file 1 — Supplementary information [file 41467_2018_5901_MOESM1_ESM.pdf]

SUPPLEMENTARY INFORMATION

Microbiota-derived short-chain fatty acids promote Th1 cell IL-10 production to maintain intestinal homeostasis

Sun et al

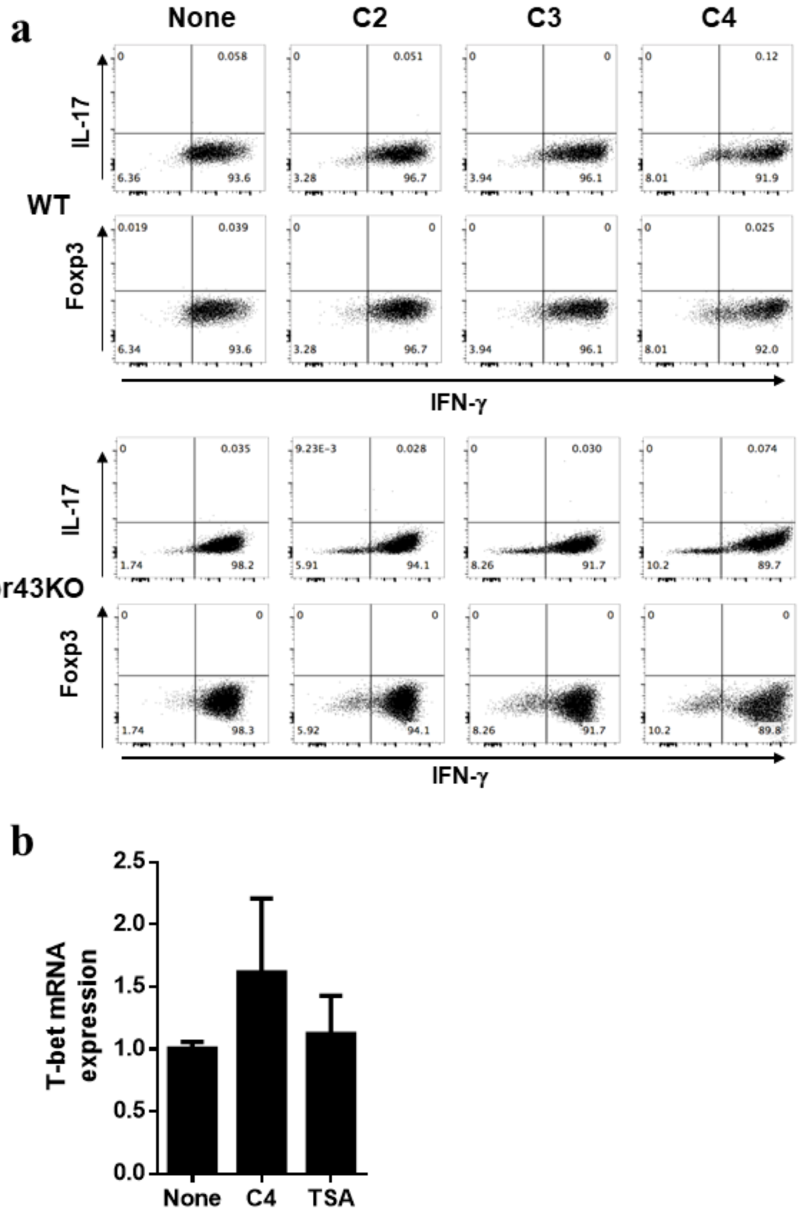

Supplementary figure 1. SCFAs do not affect expression of IL-17, Foxp3, and T-bet in Th1 cells. (a) WT and Gpr43KO CBir1 Tg Th1 cells were cultured with APC and CBir1 peptide in the presence or absence of C2, C3, and C4 for 5 days. The expression of IL-17 and Foxp3 was determined by flow cytometry analysis. (b) CBir1 Tg Th1 cells were cultured with APC and CBir1 peptide in the presence or absence of C4 and TSA for 5 days. The expression of T-bet was examined by qRT-PCR analysis. The results are shown as mean ± SD of triplicates. The data are representative of three independent experiments.

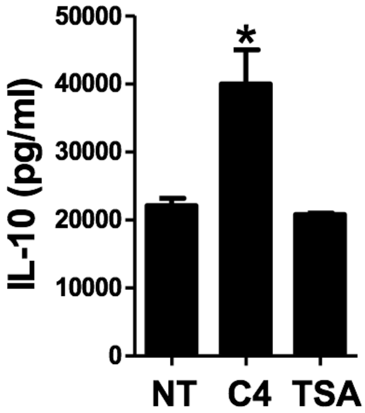

Supplementary figure 2. Butyrate but not HDAC inhibitor promotes Th1 cell production of IL-10. 1×10^6 CBir1 Th1 cells were stimulated with C4 and HDAC inhibitor TSA, respectively, for 5 days. The IL-10 in culture supernatants was determined by ELISA. Results are shown as mean ± SD of triplicates from one representative of three experiments performed. *$p<0.05$ one-way ANOVA test.

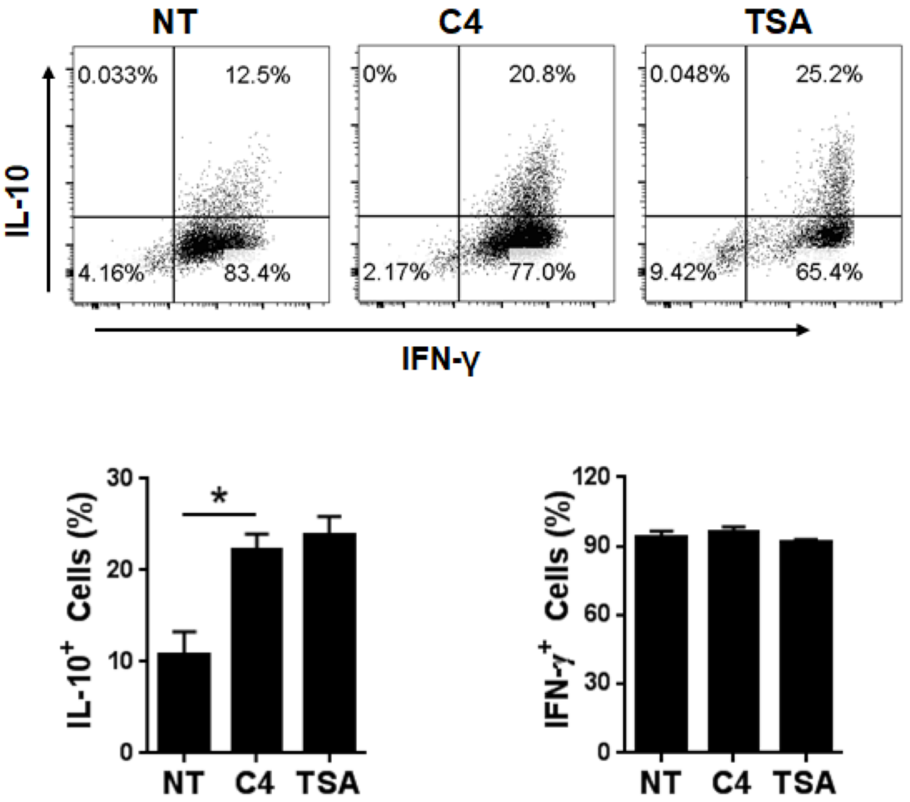

Supplementary figure 3. HDAC inhibitor promotes naïve T cell production of IL-10. 1×10^6 naïve CBir1 T cells were stimulated with C4 and HDAC inhibitor TSA, respectively, for 5 days. The expression of IL-10 and IFN-γ was examined by flow cytometry analysis. Results are shown as mean ± SD of triplicates from one representative of two experiments performed. *$p<0.05$ one-way ANOVA test.

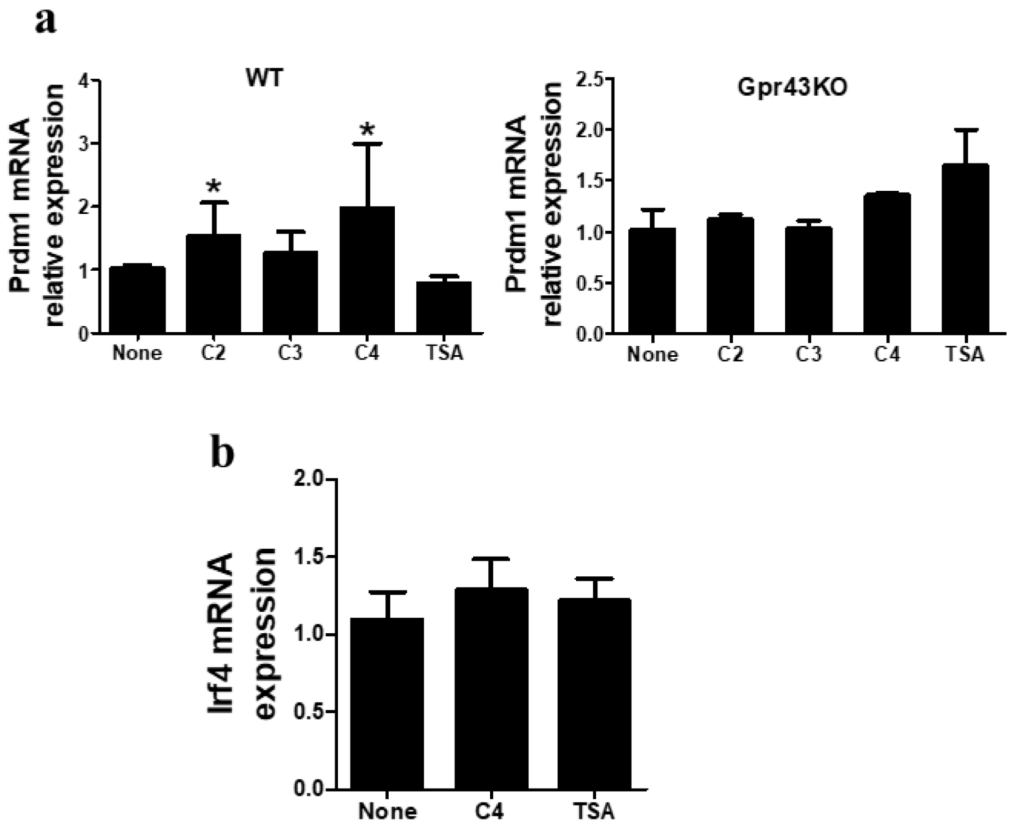

Supplementary figure 4. SCFAs promote Blimp1 expression of Th1 cells through GPR43 but not HDAC inhibition. (a) WT and GPR43KO CBir1 Tg Th1 cells were cultured with APC and CBir1 peptide in the presence or absence of C2, C3, C4, and TSA for 5 days. The expression of Blimp1 was determined by qRT-PCR. (b) CBir1 Tg Th1 cells were cultured with APC and CBir1 peptide in the presence or absence of C4 and TSA for 5 days. The expression of IRF4 was determined by qRT-PCR. The results are shown as mean ± SD of triplicates. The data are representative of three independent experiments. *p<0.05.

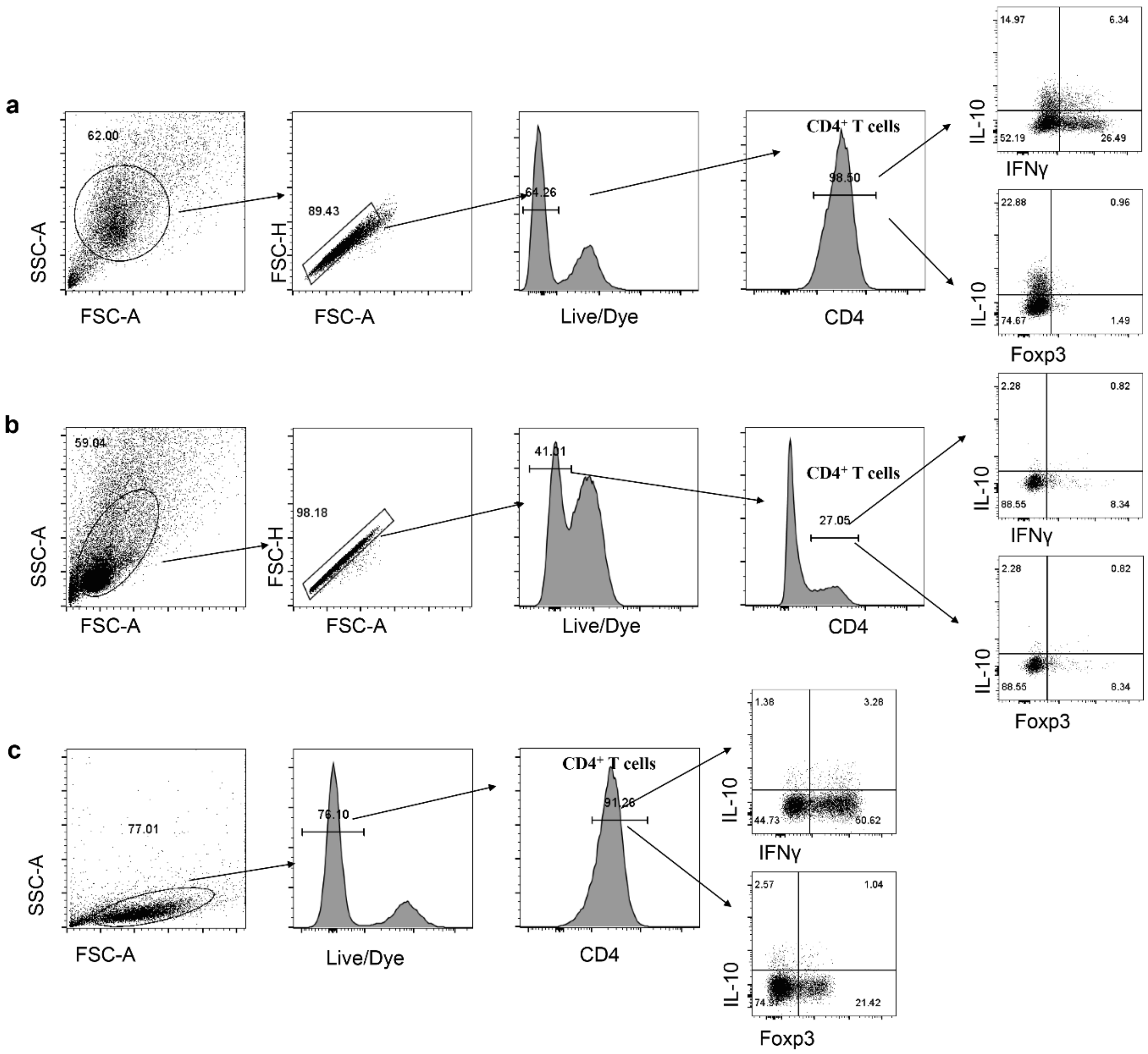

Supplementary Figure 5. FACS gating strategies. (a) Strategies to gate cytokines of mouse CD4$^+$ T cells in vitro used in Figure 2a-d, 5c-f, and 7f. (b) Strategies to gate cytokines of mouse CD4$^+$ T cells in vivo used in Figure 1c, 3a-e, 4c, 6c, and 9c. (7) Strategies to gate cytokines of human CD4$^+$ T cells in vitro used in Figure 8a.

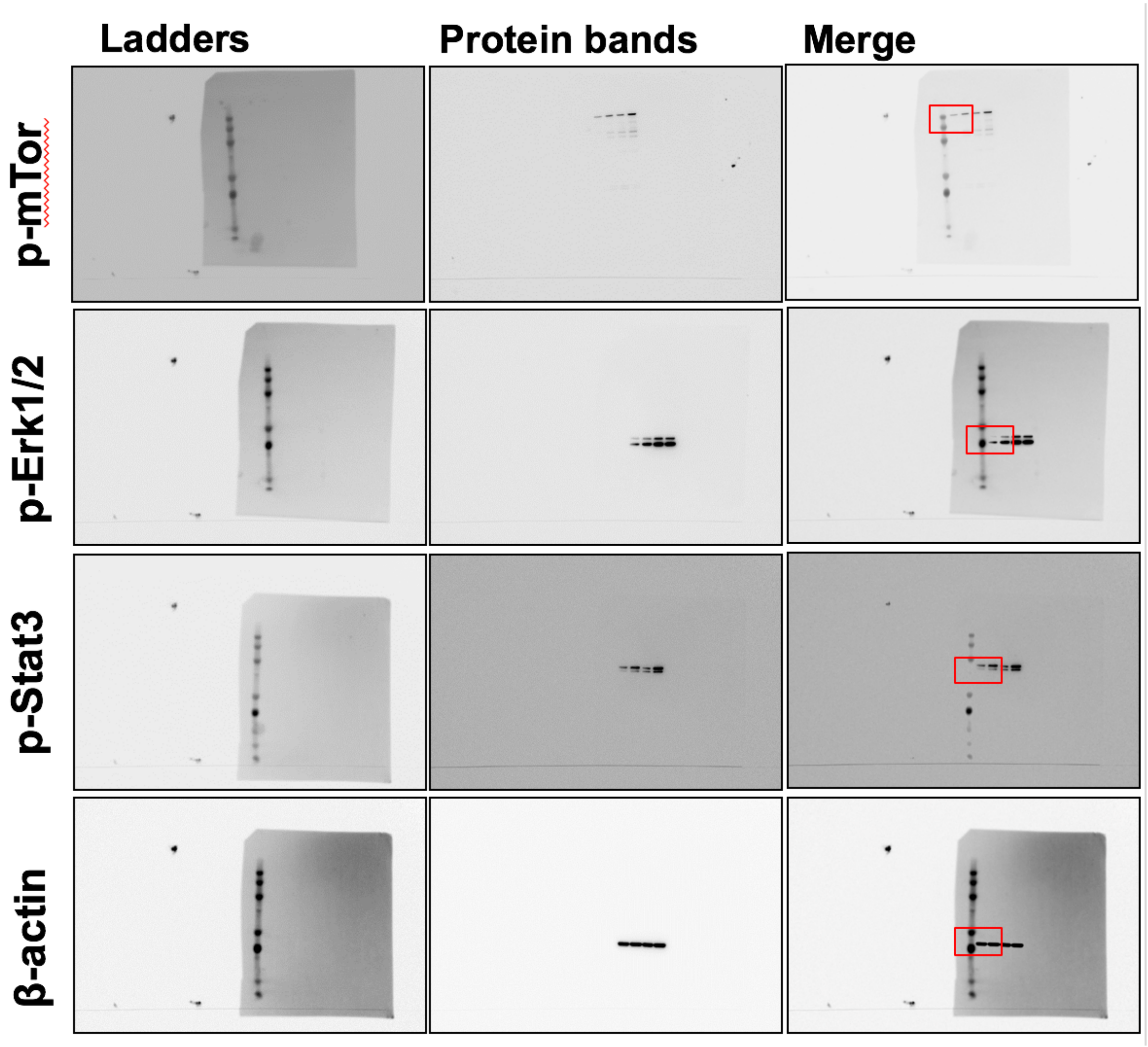

Supplementary figure 6. Uncropped scans of western blot. Th1 cells were cultured with anti-CD3 and anti-CD28 mAbs in the presence or absence of butyrate (C4). The cell lysates were prepared, and Western blot was used to determine p-mTOR, p-ERK1/2, and p-SATA3, and β-actin 15 min and 2 hrs after stimulation. Ladders and protein bands were developed using fluorescence (left panel) and chemiluminescence (middle panel), respectively. Photoshop (CC2017) was used to merge ladders and protein bands together into one picture (right panel). Cell lysates from left to right are Ctrl (15 min), C4 (15 min), Ctrl (2 hrs), and C4 (2 hrs).

Supplementary Table 1. Clinical characteristics of IBD patients

	Blood samples		
	Con	CD (A)	UC (A)
Number of patients	6	6	6
Age (y)	32.6±15.6	31.2±14.5	40.5±16.5
Gender			
Male	3	4	4
Female	3	2	2
Disease duration (month)		66±31.5	38±12.8
Current therapy			
5-aminosalicylates		4	5
Azathioprine		2	3
Corticosteroids		2	3
IFX		3	2
Disease extent (UC)			
E1			0
E2			3
E3			3
Disease location			
L1		1	
L2		2	
L3		3	
L4		0	
CRP (mg/l)		26.8±10.3	43.6±13.4

CD = Crohn's disease; UC = ulcerative colitis; IFX = infliximab; CRP = C-reactive protein.

Supplementary Table 2. Primers for real-time PCR and CRISPR-guide RNA oligo sequences

Probes and primers (ThermoFisher)			
**Genes**	**Species**	**IDs**	
GAPDH	Mouse	Mm99999915_g1	
IL-10	Mouse	Mm00439614_m1	
IL-17A	Mouse	Mm00439618_m1	
Prdm1	Mouse	Mm00476128_m1	
IFN-γ	Mouse	Mm01168134_m1	
Tbet	Mouse	Mm00450960_m1	
Ifr4	Mouse	Mm00516431_m1	
**SYBR Green Primers**			
**Genes**	**Species**	**Forward primer**	**Reverse primer**
GAPDH	Mouse	CCATGGAGAAGGCTGGGG	CAAGTTGTCATGGGATAACC
STAT3	Mouse	GGATCGCTGAGGTACAACCC	GTCAGGGGTCTCGACTGTCT
MTOR	Mouse	AGCAACAGTGAGAGTGAA	AGGAGATAGAACGGAAGAAG
MERK	Mouse	TGGGCACGAGATCCTACATG	TGGCATCAGGAGGAGGAATG
GAPDH	Human	CAGGAGGCATTGATGATGAT	GAAGGCTGGGGCTCATTT
Prdm1	Human	AAGCAACTGGATGCGCTATGT	GGGATGGGCTTAATGGTGTAGAA
**Guide RNA oligo sequences for lentiCRISPR**			
**Genes**	**Species**	**Forward primer**	**Reverse primer**
STAT3	Mouse	CACCGCGATTACCTGCACTCGCTTC	AAACGAAGCGAGTGCAGGTAATCGC
MTOR	Mouse	CACCGTGATACGAACTAGCTCGTTG	AAACCAACGAGCTAGTTCGTATCAC
ERK1	Mouse	CACCGCTTGTGCTTCTCCCGAAGAT	AAACATCTTCGGGAGAAGCACAAGC
