# Supplementary material for: Microbiota-derived short-chain fatty acids promote Th1 cell IL-10 production to maintain intestinal homeostasis
Source: Nat Commun. 2018 Sep 3;9:3555. doi: 10.1038/s41467-018-05901-2 (PMC6120873; doi:10.1038/s41467-018-05901-2)
Supplement: Supplementary file 2 — Peer Review [file 41467_2018_5901_MOESM2_ESM.pdf]

Reviewers' comments:

Reviewer #1 (Remarks to the Author):

The manuscript by Sun et al., provide data showing that short-chain fatty acids produced by intestinal microbiota act through GPR43 to induced T cells producing IL-10 and that these T cells can ameliorate a murine model of colitis. In addition, the MS contains data showing that the IL-10 production depends on activation of the mTor and Stat3 signaling that culminates in the production of Blimp-1. Finally, the MS contains data showing that human T cells respond to butyrate with increased generation of both of IL-10 producing cells and Foxp3 positive cells.

Comments:

1.The findings reported in this MS are quite similar to those previously reported by Park et al. (Mucosal Immunol. 2015, 8:80). In the latter work the ability of short-chain fatty acids (SCFA) to induce naïve T cells undergoing differentiation in Th1 or Th17 cells to produce IL-10 via activation of the mTor/Stat3 signaling was also shown as was the ability of these T cells to ameliorate gut inflammation, in this case inflammation induced in the cell transfer model of colitis or by Citrobactor rodentium. One major difference between the present study and the Park study is that induction of IL-10 producing cells by SCFA in already differentiated Th1 cells does not involve histone deacetylase (HDAC) inhibition by SCFA and depends on GPR4-recognition of SCFA on the cell surface whereas induction of IL-10 producing cells in naïve T cells undergoing T cell differentiation is dependent on short-chain fatty acid inhibition of HDAC inhibition and independent of GPR43. Since induction of IL-10 producing cells in the two situations involves the same signaling pathway (mTor/Stat3) the present data implies that GPR43 signaling via SCFA by-passes the effect HDAC inhibition and that Th1 cells are no longer susceptible to SCFA-mediated HDAC inhibition. This does not seem very likely since SCFA inhibition of HDACs is not likely to become inactive simply as a result of cell differentiation and SCFA can penetrate the cell membrane. Given that fact that the major finding in the present study is that induction of GPR43-positive IL-10 producing T cells are capable of mediating control of gut inflammation, the authors of this report need to present additional data showing SCFA-induction of IL-10 production in Th1 cells is truly independent of HDAC inhibition. This would include studies directly showing differences in responses of naïve cells undergoing differentiation and Th1 cells with respect to HDAC inhibition as well as more extensive studies showing that GPR43-deficient cells are not susceptible to HDAC inhibition by SCFA.

2.The data shown in Figure 7 relating to SCFA-induction of IL-10 production in human cells are somewhat difficult to explain. First, whereas SCFA induces IL-10-producing cells in control and IBD patients to about the same extent (Figure 7A) these regulatory cells do not have any effect on suppressing induction of IFN-g producing cells in the patient cells; in fact, SCFA appears to enhance the number of IFN-g producing cells to some extent. Since IFN-g producing cells can mediate colitis in the absence of IL-17, this result suggests that SCFA induction of IL-10-producing cells would not ameliorate IBD-related inflammation. In addition, this result conflicts with those in Figure 1 that shows that the lack of SCFA signaling in GPR43 KO cells has no effect on IL-17-producing cells and a marginal effect on IFN-g-producing cells. Second, since SCFA signaling enhances induction of Foxp3-expressing cells, it seems possible that the IL-10 producing cells are Foxp3+ cells. Dual flow cytometric studies are necessary to examine this possibility.

3.The thrust of these studies is that SCFA, in that it induces regulatory T cells, is a potential treatment of IBD. To substantiate this possibility these studies should include in vivo data showing that administration of SCFA does in fact prevent and treat colitis in a murine model of colitis. In addition, the Discussion section should confront the fact that short-chain fatty acid administration has not been notably successful as a treatment of IBD, although it may have some marginal effects in ulcerative colitis.

Reviewer #2 (Remarks to the Author):

Sun and colleagues, in the manuscript entitled "Microbiota metabolites short-chain fatty acids promote Th1 cell IL-10 production to maintain intestinal homeostasis" suggest a novel mechanism for action for short-chain fatty acids (SCFAs) in maintaining intestinal homeostasis. In short, the authors show that SCFA treatment promotes IL10 production from Th1-polarized CD4 T cells through GPR43. Treatment of Th1 polarized cells with SCFA could then limit their potential to induce colitis through induction of IL10. Although this finding has the potential to be very interesting, we have a number of comments that would strengthen the authors claims. Overall, we believe that, pending major revision, this manuscript could be acceptable for publication in Nature Communications.

Main Points
The model used through-out this study involves the in vitro polarization of CBir1 transgenic CD4+ T cells under Th1-inducing conditions prior to their transfer into lymphodeficient recipients for assessment of colitis severity and production of IL10 induction. Although the data are convincing, the main concern is that the experimental system employed is rather artificial. Additionally, we suggest the authors address the following concerns in order to clarify and strengthen the conclusions they have presented:

" The significance of IL10 producing Th1 cells in an in vivo setting remains unclear. The authors should assess whether IL10 producing Th1 cells are naturally induced when naïve CBir1Tg T cells are transferred into Rag-/- mice as well as the contribution of IL10 production by Th1 cells as compared to other IL10 producing cells in the same context.
" The authors may also consider whether IL10 producing Th1 cells are increased in vivo when naive CBirTg CD4T cells are transferred into Rag1ko mice that are treated with SCFA in their drinking water or via enema.
" Authors may also consider assessing whether IL10 producing Th1 cells are present in other in vivo models, such as during DSS-induced colitis.
" It would be pertinent to determine if in vivo treatment with SCFA is capable of increasing IL10 production by Th1 cells rather than the exclusively in vitro assessment that has been presented. The conclusion that SCFA are involved in vivo relies heavily on the assumption that deletion of Gpr43 exclusively affects the SCFA-sensing capabilities of these T cells.
" The quantification of pathological score is perplexing. As listed within the methods, 3 criteria, each with a categorical scale of 0-3 were used. This would suggest that in the most severe pathological case a score of 9 would result; however, as seen within the figures, the overall pathology scores often reach a values of 10 or above, with certain replicates - as determined by assessment of the error bars - having a value of 11 or greater. This must be corrected. Additionally, the statistical test used to compare pathological scores, must be consistent with the comparison of categorical data. There is no indication that the appropriate statistical test was employed.
" Overall the introduction would benefit from editing. The authors' tense and voice appear to shift throughout.

Additional Technical points
" Fig 1A: Magnification should be the same on all histology slides and scales bars should be added.
" Some of the differences appear to be minimal, as in they would not normally be calculated as statistically significant - a better description of the precise statistical test employed within each figure legend should be included.
" In all figure legends, number of mice per group, per experiment should be listed, as opposed to

"8 mice total combined over 3 experiments." The observed degree of variability within the presented plots may be due to pooling of individual experiments. It would be beneficial to see the differences seen within each individual experiment to determine if significance was reached within each individual case.

" Additionally, it would helpful to present the data as individual dots per animal so the reader can more accurately assess the level of variability as represented by the spread of the data.

" Figure 1C should also show FoxP3 vs IL10 on all plots to ensure Tregs are not induced after transfer.

" Figure 2A looks to represent a mild GPR43 independent induction of IL10. Are these FACs plots representative?

" Figure 2C scale bar cannot be broken in this instance - the same incrimination as the lower half of the graph is used, yet simply spread over a greater distance. Additionally, all graphs should start at zero, as the data maybe otherwise appear misleading. In the case of Figure 2C, the effect actually looks minimal from 23000 - 26000 pg/mL.

" Figure 3 - Th1 cells seem to be moving towards an increase in IL17 producing cells in GPR43KO mice. The representative plots in C are not representative of the bar graphs.

" Fig 4: It is unclear whether IL-10 producing cells are induced Tregs, which can also produce IFNg under certain conditions, in a manner also controlled by Blimp1.

" Figure 4D: appears to potentially be an induction of IL10 compared with control in Prdm1KO treated with C4

" Figure 5: The authors should provide an explanation for the reduced frequency of IFNg+ cells despite an increased level of IFNg as detected by ELISA. This suggests that overall IFNg levels are not controlled Th1 cels - what additional cellular sources could account for the observed increase in IFNg? Perhaps innate sources of IFNg should be assessed.

Reply to comments of Reviewer #1:

(1) *“The findings reported in this MS are quite similar to those previously reported by Park et al. (Mucosal Immunol. 2015, 8:80). In the latter work the ability of short-chain fatty acids (SCFA) to induce naïve T cells undergoing differentiation in Th1 or Th17 cells to produce IL-10 via activation of the mTor/Stat3 signaling was also shown as was the ability of these T cells to ameliorate gut inflammation, in this case inflammation induced in the cell transfer model of colitis or by Citrobactor rodentium. One major difference between the present study and the Park study is that induction of IL-10 producing cells by SCFA in already differentiated Th1 cells does not involve histone deacetylase (HDAC) inhibition by SCFA and depends on GPR4-recognition of SCFA on the cell surface whereas induction of IL-10 producing cells in naïve T cells undergoing T cell differentiation is dependent on short-chain fatty acid inhibition of HDAC inhibition and independent of GPR43. Since induction of IL-10 producing cells in the two situations involves the same signaling pathway (mTor/Stat3) the present data implies that GPR43 signaling via SCFA by-passes the effect HDAC inhibition and that Th1 cells are no longer susceptible to SCFA-mediated HDAC inhibition. This does not seem very likely since SCFA inhibition of HDACs is not likely to become inactive simply as a result of cell differentiation and SCFA can penetrate the cell membrane. Given that fact that the major finding*

in the present study is that induction of GPR43-positive IL-10 producing T cells are capable of mediating control of gut inflammation, the authors of this report need to present additional data showing SCFA-induction of IL-10 production in Th1 cells is truly independent of HDAC inhibition. This would include studies directly showing differences in responses of naïve cells undergoing differentiation and Th1 cells with respect to HDAC inhibition as well as more extensive studies showing that GPR43-deficient cells are not susceptible to HDAC inhibition by SCFA."

Response: We appreciate the reviewer's excellent comments. As pointed by the reviewer, our data demonstrated that SCFAs not only promoted naïve T cell differentiation and production of IL-10, but also promoted differentiated Th1 cell production of IL-10, which inhibits intestinal inflammation induced by Th1 cells in response to gut microbiota antigens, thus contributes to maintenance of intestinal homeostasis. As intensive investigations have been carried out on the regulation of naïve T cell differentiation, how T effector cells are regulated following differentiation, which represents the T cells in the inflamed lesions in many autoimmune diseases as well as in IBD, is relatively unclear. Our study thus offers a novel mechanism by which gut microbiota regulates intestinal immune homeostasis through production of metabolites short chain fatty acids. However, different from SCFA regulation of naïve T cell IL-10 production, which is mediated by HDAC inhibitory activity, our data indicated that SCFAs promotion of differentiated Th1 cell IL-10 production is mainly mediated by GPR43 but not HDAC inhibitory activity. Among many possibilities behind the different mechanisms by which SCFAs regulate IL-10 production in naïve T cell vs differentiated Th1 cells, naïve T cells express GPR43 at a very low level whereas differentiated Th1 cells express high levels of GPR43. We speculate that strong activation of GPR43 by SCFAs in differentiated Th1 cells may mask the effect of HDAC inhibitory activity, while HDAC inhibitory activity is dominant in SCFA regulation of naïve T cells.

As suggested by the reviewer, we performed the experiments directly comparing IL-10 production by naïve T cells undergoing differentiation and differentiated Th1 cells with respect to HDAC inhibition, as well as the experiments on GPR43-deficient T cells in response to HDAC inhibition by SCFA. Our data demonstrated that HDAC inhibitor induced IL-10 production by naïve T cells undergoing differentiation but not by differentiated Th1 cells when both groups were done side by side (**Fig 2c-d and Supplementary Fig 3**). Our data also indicated that GPR43-deficient cells are not susceptible to HDAC inhibition by SCFA in inducing differentiated Th1 cell production of IL-10. Those data are now included (**Fig 2c-d**) and also discussed in the revised manuscript (**Page 22**).

(2) "*The data shown in Figure 7 relating to SCFA-induction of IL-10 production in human cells are somewhat difficult to explain. First, whereas SCFA induces IL-10-producing cells in control and IBD patients to about the same extent (Figure 7A) these regulatory cells do not have any effect on suppressing induction of IFNγproducing cells in the patient cells; in fact, SCFA*

appears to enhance the number of IFNγ producing cells to some extent. Since IFNγ producing cells can mediate colitis in the absence of IL-17, this result suggests that SCFA induction of IL-10-producing cells would not ameliorate IBD-related inflammation. In addition, this result conflicts with those in Figure 1 that shows that the lack of SCFA signaling in GPR43 KO cells has no effect on IL-17-producing cells and a marginal effect on IFNγ-producing cells. Second, since SCFA signaling enhances induction of Foxp3-expressing cells, it seems possible that the IL-10 producing cells are Foxp3^{+} cells. Dual flow cytometric studies are necessary to examine this possibility."

Response: We appreciate the reviewer's excellent comments. The human T cells we used are from peripheral blood, which contain both naïve and differentiated T cells. Our data indicated that SCFA promoted human T cell IL-10 production and Foxp3 expression but did not affect IFNγ-producing T cells in both normal individuals and IBD patients. Although it is well-established that IFNγ-producing Th1 cells induce colitis, unfortunately, the machanisms involved are still unclear. IFNγ is not the major cytokine mediating colitis induction, in that blockade of IFNγ or using IFNγ-deficient or IFNγR-deficient mice do not affect colitis development much, which is the main reason behind the discovery of Th17 cells. Furthermore, clinic trials of anti-IFNγ mAbs failed in IBD patients while ago. Thus, production of IFNγ may only represents a signature marker of Th1 cell population which can induce colitis. An elegant study recently demonstrated that innate cell signaling of IL-10 was crucial in regulation T cell-mediated colitis (**Shouval DS et al Immunity 2014 40:706**). Thus, that SCFAs promote human T cell production of IL-10 but do not inhibit IFNγ production does not necessarily suggest they will not inhibit Th1 function in vivo, especially in IBD patient. Our data further demonstrated that blockade of IL-10 signaling dramatically promoted Th1 cell-induced colitis (**new Fig 4** in the revised manuscript), indicating IL-10 production by Th1 cells serves as a self-limiting factor to inhibit colitis progression. It also suggests that this is not conflict with the data in Fig 1, in that there is no much difference of T cell production of IFNγ in recipient mice of WT Th1 cells and GPR43 KO Th1 cells. The more severe colitis in recipient mice of GPR43 KO Th1 cells is most likely due to lower level of IL-10 production by GPR43 KO Th1 cells as we showed in **new Fig 4** of the revised manuscript.

As suggested by the reviewer, we analyzed the data by using parameters of IL-10 vs Foxp3. As shown in **new Fig 8** in the revised manuscript, around 50% of IL-10-positive T cells are IFNγ-positive but are Foxp3-negative in human T cells. As in mouse Th1 cells (**new Fig 2** in the revised manuscript), the Th1 cells are Foxp3-negative, and SCFA treatment induced IFNγ-positive T cells to produce IL-10 but did not promote IFNγ-positive T cells to express Foxp3. Thus, although SCFAs induce naïve T cell expression of Foxp3 during differentiation, they do not induce differentiated Th1 cell to express FoxP3.

(3) "The thrust of these studies is that SCFA, in that it induces regulatory T cells, is a potential treatment of IBD. To substantiate this possibility these studies should include in vivo data showing that administration of SCFA does in fact prevent and treat colitis in a murine model of colitis. In addition, the Discussion section should confront the fact that short-chain fatty acid administration has not been notably successful as a treatment of IBD, although it may have some

marginal effects in ulcerative colitis."

Response: We appreciate the reviewer's excellent suggestion. As suggested by the reviewer, we performed the experiments using DSS colitis model. Our data indicated that oral feeding butyrate indeed inhibited colitis development induced by DSS insults. Those data are now included in **Fig 5** of the revised manuscript. We also discuss in the revised manuscript (**Page 23**) the fact that short-chain fatty acid administration has not been notably successful as a treatment of IBD, although it may have some marginal effects in ulcerative colitis, as it is most likely due to timing, route, doses, etc.

Reply to comments of Reviewer #2:

(1) *"The model used through-out this study involves the in vitro polarization of CBir1 transgenic $CD4^+$ T cells under Th1-inducing conditions prior to their transfer into lymphodeficient recipients for assessment of colitis severity and production of IL10 induction. Although the data are convincing, the main concern is that the experimental system employed is rather artificial."*

Response: We appreciate the reviewer's comments. To make the study more relavent, we now included another animal model, i.e. DSS colitis, in which oral feeding SCFA indeed inhibited colitis, in the revised manuscript (**Fig 9**).

(2) "Additionally, we suggest the authors address the following concerns in order to clarify and strengthen the conclusions they have presented:

" The significance of IL10 producing Th1 cells in an in vivo setting remains unclear. The authors should assess whether IL10 producing Th1 cells are naturally induced when naïve CBir1Tg T cells are transferred into $Rag^{-/-}$ mice as well as the contribution of IL10 production by Th1 cells as compared to other IL10 producing cells in the same context."

Response: We appreciate the reviewer's excellent suggestion. We performed the experiments as suggested by the reviewer. Our data indicated that when transferred into $Rag^{-/-}$ mice, naïve CBir1Tg T cells differentiate into all subsets of T cells, insluding Th1, Th17, and Treg as identified by expression of IFNγ, IL-17, and Foxp3 respectively. Although all T cell subsets can produce IL-10, Th1 cells are actually dominant producers of IL-10 among all T cell subsets. Our data further demonstrated that blockade of IL-10 signaling dromatically promoted Th1 cell-induced colitis (**new Fig 4** in the revised manuscript), indicating that Th1 cells may not only serve as inducer of colitis but also serve as a brake for colitis progression once IL-10 is produced. Those data are inluded in the revised manuscript (**Fig 3a**).

(3) "The authors may also consider whether IL10 producing Th1 cells are increased in vivo when naive CBirTg CD4 T cells are transferred into Rag1ko mice that are treated with SCFA in their drinking water or via enema."

Response: We performed the experiments as suggested by the reviewer. Our data indicated that oral feeding SCFA in drinking water promoted IL-10 production by naïve CBir1Tg T cells when transferred into Rag1ko mice. Those data are inluded in the revised manuscript (**Fig 3b and c**).

(4) " Authors may also consider assessing whether IL10 producing Th1 cells are present in other in vivo models, such as during DSS-induced colitis."

Response: We performed the experiments as suggested by the reviewer. Our data indicated that IL10 producing Th1 cells are also present in model of DSS-induced colitis. Furthermore, oral feeding SCFA inhibited DSS colitis. Those data are included in the revised manuscript (**Fig 9a-c**).

(4) "It would be pertinent to determine if in vivo treatment with SCFA is capable of increasing IL10 production by Th1 cells rather than the exclusively in vitro assessment that has been presented. The conclusion that SCFA are involved in vivo relies heavily on the assumption that deletion of Gpr43 exclusively affects the SCFA-sensing capabilities of these T cells."

Response: We performed the experiments as suggested by the reviewer. Our data indicated that SCFA indeed promoted IL10 production by differentiated Th1 cells in vivo when transferred into Rag-/- mice. Those data are included in **Fig 3d and e** of the revised manuscript .

(5) "The quantification of pathological score is perplexing. As listed within the methods, 3 criteria, each with a categorical scale of 0-3 were used. This would suggest that in the most severe pathological case a score of 9 would result; however, as seen within the figures, the overall pathology scores often reach a values of 10 or above, with certain replicates - as determined by assessment of the error bars - having a value of 11 or greater. This must be corrected. Additionally, the statistical test used to compare pathological scores, must be consistent with the comparison of categorical data. There is no indication that the appropriate statistical test was employed."

Response: We appreciate the reviewer's careful reading through our manuscript and apologize for not carefully describing the quantification of pathological score. We actually quantified the severity of tissue damage by using 5 criteria, each with a categorical scale of 0-3, i.e. hyperplasia; goblet cell number; crypt abscesses; ulceration; mucosa and submucosa inflamamtory cell infiltration.This has been corrected in the revised manuscript (**Page 10**).

(6) "Overall the introduction would benefit from editing. The authors' tense and voice appear to shift throughout."
Response: The introduction has been modified intensively in the revised manuscript.

(7) “Additional Technical points
" Fig 1A: Magnification should be the same on all histology slides and scales bars should be added.
" Some of the differences appear to be minimal, as in they would not normally be calculated as statistically significant - a better description of the precise statistical test employed within each figure legend should be included.
" In all figure legends, number of mice per group, per experiment should be listed, as opposed to "8 mice total combined over 3 experiments." The observed degree of variability within the presented plots may be due to pooling of individual experiments. It would be beneficial to see the differences seen within each individual experiment to determine if significance was reached within each individual case.
" Additionally, it would helpful to present the data as individual dots per animal so the reader can more accurately assess the level of variability as represented by the spread of the data.”

Response: Done accordingly.

(8) " Figure 1C should also show FoxP3 vs IL10 on all plots to ensure Tregs are not induced after transfer.”

Response: Done accordingly. See **new Fig 1c** in the revised manuscript. Tregs are not induced after transfer.

(9) "Figure 2A looks to represent a mild GPR43 independent induction of IL10. Are these FACs plots representative?”

Response: We appreciate the reviewer’s careful reading through out data. A more representive FACs plots profile is shown in new **Fig 2a** of the revised manuscript.

(10) "Figure 2C scale bar cannot be broken in this instance - the same incrimination as the lower half of the graph is used, yet simply spread over a greater distance. Additionally, all graphs should start at zero, as the data maybe otherwise appear misleading. In the case of Figure 2C, the effect actually looks minimal from 23000 - 26000 pg/mL.”

Response: Done accordingly.

(11) "Figure 3 - Th1 cells seem to be moving towards an increase in IL17 producing cells in GPR43KO mice. The representative plots in C are not representative of the bar graphs.”

Response: See **new Fig 4c and d** in the revised manuscript. We carefully checked both bar graphs and the plots, and the plots showed an increase in $IL17^{+}$ $IFN\gamma^{+}$ T cells in GPR43KO mice, which resulted in an increase of total IL-17 producing cells as showed in **bar charts of Fig 4d**.

(12) "Fig 4: It is unclear whether IL-10 producing cells are induced Tregs, which can also produce IFNg under certain conditions, in a manner also controlled by Blimp1."

Response: We added FACS data of Foxp3 vs IL-10 in **new Fig 5e** in the revised manuscript, which showed that C4 did not induce IL-10 in Foxp3-positive cells.

(13) "Figure 4D: appears to potentially be an induction of IL10 compared with control in Prdm1KO treated with C4".

Response: See **new Fig 5d.** We did statistical analysis on IL-10 production in Prdm1 KO Th1 cells treated with or without C4. The increase of IL-10 by C4 is not significant (**P=0.287**).

(14) "Figure 5: The authors should provide an explanation for the reduced frequency of $IFN\gamma^{+}$ cells despite an increased level of IFNγ as detected by ELISA. This suggests that overall IFNγ levels are not controlled Th1 cells - what additional cellular sources could account for the observed increase in IFNγ? Perhaps innate sources of IFNγ should be assessed.

Response: We carefully checked our data in original Figure 5 (**new Fig 6** in the revised manuscrit), level of IFNγ in recipient mice of SCFA-treated WT Th1 cells was actually decreased as detected by ELISA compared to recipient mice of WT Th1 cells. The level of IFNγ in recipient mice of SCFA-treated $Blimp\text{-}1^{-/-}$ Th1 cells was similar to that in recipient mice of $Blimp\text{-}1^{-/-}$ Th1 cells.

REVIEWERS' COMMENTS:

Reviewer #1 (Remarks to the Author):

The authors have addressed the several concerns I raised in my initial review. The fact remains, however, that administration of short chain fatty acids have not proven to be a successful treatment of IBD and this is not likely to be due simply to technical difficulties relating to how the SCF were administered.

Another misconception of the authors is that IFN-g is not an important pro-inflammatory cytokine in IBD. Blockade of IFN-g does result in reduced inflammation in animal models and it is likely that IFN-g arising from Th17 cells is in fact the main mediator of IBD.

Reviewer #2 (Remarks to the Author):

The authors have satisfactorily addressed many of our criticisms; however, minor edits should still be made prior to finalizing this manuscript for publication. In doing so, we suggest the authors consider the following additional comments:

We advise that the authors avoid making overly generalized statements, such as: “it is still largely unclear how effector T cells are regulated.” A very large body of literature exists on the regulation of effector T cells.

As written, some sentences are rather confusing. For example, “around 50% of IL10 producing cells were IFNg+ Th1 cells, indicating that Th1 cells are dominant producers of IFNg amongst all T cell subsets.” This sentence is unclear; Th1 cells are routinely defined by their production of IFNg. Furthermore, if the authors intended to write “…indicating that Th1 are dominant producers of IL-10…” then it would be pertinent to qualify this sentence by reiterating that this phenotype is observed following transfer into RagKO mice. Additionally, the gating strategy used to identify IFNg+IL-10+ cells within Figure 3 (and later figures describing ex vivo analysis) is not very robust; the gate appears to be incorrectly placed and could be overinflating the frequency of this population. The authors may consider including their entire gating strategy and/or isotype controls as supplementary data to confirm that this staining and gating are truly representative of IL-10 production by this IFNg+ population.

Reply to comments of Reviewer #1:

(1) *"administration of short chain fatty acids has not proven to be a successful treatment of IBD and this is not likely to be due simply to technical difficulties relating to how the SCF were administered."*

Response: We agree with the reviewer that administration of short chain fatty acids have not proven to be a successful treatment of IBD, although it may have some effects in ulcerative colitis, which requires further investigation. Among many possible reasons, as short chain fatty acids are readily absorbed by the epithelial cells in the intestinal track, an appropriate dose would be crucial. It has been reported that SCFA mixtures enemas and butyrate enemas had better beneficial effects in patients with UC (Scheppach W, Sommer H, Kirchner T, Paganelli GM, Bartram P, Christl S, et al. Effect of butyrate enemas on the colonic mucosa in distal ulcerative colitis. Gastroenterology 1992;103:51-566; Hamer, HM et al Clinical Nutrition 29 (2010) 738-744), an appropriate route should also be considered. More importantly, understanding the mechanisms by which SCFAs regulate IBD will provide insights into how to use SCFAs in treatment of IBD. This part is included in discussion of the manuscript.

(2) "*Another misconception of the authors is that IFNγ is not an important pro-inflammatory cytokine in IBD. Blockade of IFN-g does result in reduced inflammation in animal models and it is likely that IFN-g arising from Th17 cells is in fact the main mediator of IBD.*"

Response: We agree with the reviewer that IFNγ is an important pro-inflammatory cytokine in animal models of colitis. However, anti-IFNγ antibody has not been successful in treatment of IBD patients, indicating that more complicated role of IFNγ in IBD.

Reply to comments of Reviewer #2:

(1) *"We advise that the authors avoid making overly generalized statements, such as: "it is still largely unclear how effector T cells are regulated." A very large body of literature exists on the regulation of effector T cells."*

Response: We appreciate the reviewer's comments. It has been changed to "it is still not completely clear" in the revised manuscript (**page 4**).

(2) "*As written, some sentences are rather confusing. For example, "around 50% of IL10 producing cells were IFNg+ Th1 cells, indicating that Th1 cells are dominant producers of IFNg amongst all T cell subsets." This sentence is unclear; Th1 cells are routinely defined by their production of IFNg. Furthermore, if the authors intended to write "…indicating that Th1 are dominant producers of IL-10…" then it would be pertinent to qualify this sentence by reiterating that this phenotype is observed following transfer into RagKO mice.*"

Response: We have modified accordingly in the revised manuscript (**Page 9**).

UNIVERSITY OF TEXAS MEDICAL BRANCH ROOM 4.142C MEDICAL RESEARCH BUILDING
301 UNIVERSITY BOULEVARD GALVESTON, TEXAS 77555-1019
(409) 772-4902 FAX (409) 772-5065
e-mail yicong@utmb.edu

(3) "*Additionally, the gating strategy used to identify IFNg+IL-10+ cells within Figure 3 (and later figures describing ex vivo analysis) is not very robust; the gate appears to be incorrectly placed and could be overinflating the frequency of this population. The authors may consider including their entire gating strategy and/or isotype controls as supplementary data to confirm that this staining and gating are truly representative of IL-10 production by this IFNg+ population*."

Response: The IL-10 gating strategy in now included as **Supplementary Fig 5** in the revised manuscript.

UNIVERSITY OF TEXAS MEDICAL BRANCH ROOM 4.142C MEDICAL RESEARCH BUILDING
301 UNIVERSITY BOULEVARD GALVESTON, TEXAS 77555-1019
(409) 772-4902 FAX (409) 772-5065
e-mail yicong@utmb.edu
